# Prediction of Epitope-Based Peptides for the Utility of Vaccine Development from Fusion and Glycoprotein of Nipah Virus Using *In Silico* Approach

**DOI:** 10.1155/2014/402492

**Published:** 2014-07-24

**Authors:** M. Sadman Sakib, Md. Rezaul Islam, A. K. M. Mahbub Hasan, A. H. M. Nurun Nabi

**Affiliations:** ^1^Department of Biochemistry and Molecular Biology, University of Dhaka, Dhaka 1000, Bangladesh; ^2^International Max Planck Research School for Neurosciences, University of Göttingen, 37077 Göttingen, Germany

## Abstract

This study aims to design epitope-based peptides for the utility of vaccine development by targeting glycoprotein G and envelope protein F of Nipah virus (NiV) that, respectively, facilitate attachment and fusion of NiV with host cells. Using various databases and tools, immune parameters of conserved sequence(s) from G and F proteins of different isolates of NiV were tested to predict probable epitope(s). Binding analyses of the peptides with MHC class-I and class-II molecules, epitope conservancy, population coverage, and linear B cell epitope prediction were analyzed. Predicted peptides interacted with seven or more MHC alleles and illustrated population coverage of more than 99% and 95%, for G and F proteins, respectively. The predicted class-I nonamers, SLIDTSSTI and EWISIVPNF, superimposed on the putative decameric B cell epitopes, were also identified as core sequences of the most probable class-II 15-mer peptides GPKVSLIDTSSTITI and EWISIVPNFILVRNT. These peptides were further validated for their binding to specific HLA alleles using *in silico* docking technique. Our *in silico* analysis suggested that the predicted epitopes, either GPKVSLIDTSSTITI or EWISIVPNFILVRNT, could be a better choice as universal vaccine component against NiV irrespective of different isolates which may elicit both humoral and cell-mediated immunity.

## 1. Introduction

Nipah virus (NiV), first reported in Malaysia in 1998 mainly causing nonlethal respiratory disease in pigs, is a nonsegmented single stranded linear negative sense RNA pathogenic paramyxovirus virus [1, 2]. In humans, 105 deaths were reported in Malaysia mainly due to inflammation of the brain (encephalitis) or respiratory diseases [2]. Initially, it was considered as the zoonotic virus and NiV infection was prevalent among pig farmers, pork sellers, and army personnel involved in the culling of pigs in Malaysia. Pigs were supposed to be the amplifying host, which were believed to be infected through fruits contaminated by body secretions and/or body fluids of bats [2]. However, patients who have never come into contact with pigs have been found to be infected with NiV, thus implying direct transmission from fruit bat. Person-to-person transmission of this virus has also been manifested in Bangladesh [3, 4]. Though many patients recover fully, the mortality rate should be taken into consideration. In different districts of Bangladesh, around 50% mortality rate of the NiV infected patients has been reported till 2011 [5]. Although the occurrences of NiV infection are limited to few countries of the world so far, the territory of the natural hosts of NiV infection (fruit bats) is widely distributed in the world [5] from Australia and Southeast and South Asia to west coast of Africa. To prevent further infection, it is indeed the necessity of time to develop effective vaccines and/or therapeutics.

Using available knowledge on immunity to other paramyxoviruses [6–10], both F (fusion) and G (glycoprotein) proteins of NiV have been chosen as the best candidate for vaccine development against this deadly virus. Recombinant NiV F and G proteins expressed in Vaccinia virus have been shown to be immunogenic by inducing protective immune responses in hamsters [7]. A recombinant subunit vaccine based on the Henipavirus attachment G glycoprotein manages to completely protect subsequent NiV infection [8]. Canarypox virus-based vaccine vectors carrying genes encoding NiV F or G proteins induce neutralizing antibodies in pigs and prevent viral shedding during NiV challenge [9]. Also, Yoneda and his group [10] reported live attenuated recombinant measles virus vaccine expressing NiV envelop glycoprotein G. However, all these vaccines could not surpass animal test and, currently, there are no vaccines licensed for human use.

Secreted and surface proteins of a pathogen are mostly antigenic and responsible for pathogenicity [11]. The envelope protein assists viral admission through host cell surface receptors and is also the primary target of B cells through immunoglobulin molecules [11], which could be considered as the good candidates for designing vaccine. Hence, B cell epitope prediction is one of the steps in vaccine designing [12]. Similarly, effective immune response depends on specificity and diversity of the antigen binding to the human leukocyte antigen (HLA) [13] class I (recognizes CD8^+^ T-cells) and class II (recognizes CD4^+^ T-cells) alleles [14, 15]. Moreover, because of the high HLA polymorphism, it is essential to recognize peptides that bind more than one HLA allele for the development of vaccines with impartial and extensive human population coverage. This eventually would help to curtail total number of predicted epitopes without negotiating the population coverage required in the design of multiepitope vaccines. Conventional techniques for vaccine development are laborious and time consuming. As a result computational methods, an alternative* in silico* models [16], for predicting epitopes have attracted attention of the researchers to reduce the cost and time of vaccine development to fight with the rapidly growing devastating organisms. Currently, several immunoinformatics tools are available for predicting B and T cell epitopes with high sensitivity and specificity. These tools are playing a vital role in understanding the molecular basis of immunity and, notably in the development of epitope based-peptide vaccines, immunotherapy against cancer and autoimmune diseases. In this study, we used some of the mostly referenced computational* in silico* methods for predicting epitope-based peptides for the utility of vaccine development against the deadly Nipah virus.

## 2. Methods

**Figure 1:**
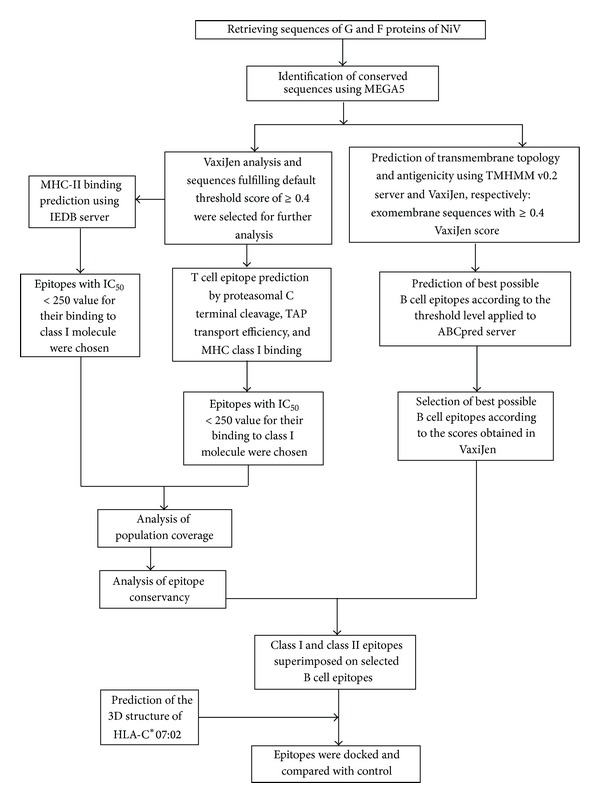
Flowchart summarizing the protocols undertaken to complete the epitope prediction.

An outline of the methodology undertaken for this study has been portrayed in Figure 1.

### 2.1. Retrieving Protein Sequences and Multiple Sequence Alignment

The sequences of glycoprotein G and fusion protein F of different isolates of NiV have been retrieved from uniprot (http://www.uniprot.org) and NCBI protein database (http://www.ncbi.nlm.nih.gov/protein) in FASTA format. These sequences were deposited in the databases obtained from different parts of NiV endemic regions such as Faridpur, Manikgonj, and Rajbari districts of Bangladesh as well as Malaysia and India at different time. The habitats of isolates include pigs and humans.

Retrieved sequences were subjected to multiple sequence alignment using MEGA 5.05 software package (http://www.megasoftware.net). The CLUSTALW algorithm along with 1000 bootstrap value and other default parameters were used to fabricate the alignment. The sequences were analyzed with a view to recognize the immunologically pertinent regions that were achieved by predicting epitopic peptides. An amino acid stretch must be of a minimum length for being considered as an epitope that we are aiming to design. Due to representative length of peptide that binds to HLA molecules, nonamers were selected as the minimum length of the conserved sequences for the prediction of epitope-based peptide in this study.

### 2.2. Prediction of Antigenicity and Transmembrane Properties of the Conserved Sequences

Recognition of the molecules by the antibodies and/or cells of the immune system are known as their antigenicity. The conserved amino acid sequences from G and F proteins were screened for predicting their antigenicity using an online antigen prediction server, VaxiJen v2.0 [17]. These sequences were tested for predicting T cell epitopes.

On the other hand, the antigenic conserved sequences were also scrutinized to distinguish their soluble and membrane parts. To perform this prediction, each selected amino acid sequence was subjected to transmembrane topology prophecy using TMHMM v0.2 server [18] in order to discriminate intracellular and surface proteins with high degree of accuracy.

### 2.3. Prediction of T Cell Epitopes from the Conserved Sequences

The cytotoxic T lymphocyte (CTL) epitopes from the conserved peptides were predicted using the NetCTL 1.2 server available at http://www.cbs.dtu.dk/services/NetCTL/ that is based on the neural network architecture. This predicts candidate epitopes based on the processing of the peptides* in vivo* [19] which also covers 12 HLA-I super types (A1, A2, A3, A24, A26 B7, B8, B27, B39, B44, B58, and B62). The sensitivity and specificity levels were, respectively, set at 0.89 and 0.94, by setting the threshold level at 0.5 during analysis. This would help to assess our findings more decisively by generating more epitopes. A combined algorithm integrating MHC class I binding, transporter of antigenic peptides (TAP) transport efficiency, and proteosomal cleavage prediction was involved to predict a total score. Based on this score, the best candidates were selected for further analysis. To calculate the IC_50_ values required for the binding of peptide molecules to the specific MHC alleles, Stabilized Matrix Method (SMM)-based prediction tool in Immune Epitope Database (IEDB) was applied. All the available MHC alleles were selected and the peptide lengths were set at 9.0 before making prediction. The parameters for immunogenicity detection (TAP score, proteasomal score, and IC_50_ values) were normalized on a scale of 0 to 1 and were given a weighted score to prioritize the parameters in order to determine the immunogenicity.

Conserved peptides were also tested for predicting epitopes that interact with MHC class II molecules by selecting all the alleles in IEDB MHC class II binding prediction tool (http://tools.immuneepitope.org/mhcii/). In this case, SMM-align method [20] was employed to find out good MHC class II candidate binders. The top scoring peptides were selected by setting cut-off values of IC_50_ for the predicted binders within 250 nM.

### 2.4. Population Coverage and Prediction of Epitope Conservancy

Prediction of T cell epitope is not enough for becoming a good candidate peptide as vaccine. It should be taken into consideration that, along with identification of MHC allele-specific T cell epitope, the predicted epitope(s) should effectively cover human population. Predicted epitopes showed interaction with different MHC alleles. To find out the human population coverage of the individual epitopes, predicted epitopic sequences with the corresponding Class I HLA alleles were submitted to the population coverage analysis tool of IEDB (http://tools.immuneepitope.org/tools/population/iedb_input) by maintaining the default analyses parameters just as it is (Population/Area = 78 populations grouped into 11 different geographical areas). For calculating the population coverage in IEDB, latest data from allelefrequencies.net database (2011 and onwards) were used, which is the most comprehensive one available because of its huge population datasets. For individual population coverage, the tool computes the followings: (i) apprehended population coverage; (ii) average number of hits by the epitopes/HLA combinations recognized by different ethnic groups or populations; and (iii) minimum number of hits by the epitopes/HLA combinations recognized by 90% of the population (PC90). These calculations were made on the basis of HLA genotypic frequencies assuming nonlinkage disequilibrium between HLA loci.

Epitope conservancy of the selected epitopes was tested using epitope conservancy tool (http://tools.immuneepitope.org/tools/conservancy/iedb_input) available in IEDB analysis resource. The conservancy level of each potential epitope was calculated by looking for identities in all 18 protein sequences of different strains retrieved from database.

### 2.5. Putative B Cell Epitope Prediction

Linear B cell epitopes are of variable lengths of peptides from 2 to 85 compared to that of T cell epitopes. Linear B cell epitopes were predicted using ABCpred prediction server (http://www.imtech.res.in/raghava/abcpred/). To do so, conserved sequences with ≥0.4 VaxiJen scores and exomembrane topology were applied in prediction server by setting cut-off value at 0.51 and the length of the epitopes was fixed as decamer. Overlapping sequences were also filtered. The nonamers which were significantly superimposed (≥7 amino acid overlaps) on putative B cell epitope (decamer peptides) were considered for interpretation.

### 2.6. Prediction of the 3D Structures of the Predicted Epitope Peptides and HLA-C 07∗02 Allele for Molecular Docking

The molecular docking of the predicted epitopic peptides was performed with the predicted structures of the respective best fitted HLA alleles. The 3D structures of the peptides and HLA alleles were also predicted. The 3D structures of the selected peptides were designed using the PEP-FOLD Peptide Structure Prediction server at the RPBS mobile portal [21, 22]. The best models provided by the server were chosen for the docking study.

The 3D structure of HLA-C 07∗02 allele was predicted using phyre2 protein prediction server (Protein Homology/analogy Recognition Engine v2.0) [23]. The cds sequence of HLA-C 07∗02 was obtained from NCBI protein database (GenBank ID: BAA08625.1 or uniprot ID: P10321) and the prediction of 3D structure was performed using template chain A of H-2kb MHC class I molecule with PDB ID: 1KJ3 that covered 76% of query sequence modeled with 100% confidence. Structural evaluation and stereochemical analyses were performed using different evaluation and validation tools. Ramachandran plot obtained from PROCHECK analysis helped to evaluate backbone conformation. The Ramachandran plot of the phi/psi distribution in the model is developed using PROCHECK [24] for checking non-GLY residues at the excluded regions. The overall model quality was validated using Z-score (determined by PROSA web tool [25]) which is used to check whether the input structure is within the range of scores typically found in native proteins of similar size. The model was further evaluated through ERRAT [26]. PyMOL graphics was used to superimpose the predicted structure of HLA-C 07∗02 with the crystal structure of HLA-C 07∗02.

### 2.7. Molecular Docking Study of HLA-Peptide Interaction

#### 2.7.1. HLA-Epitope Binding Prediction

The AutoDOCK tool from the MGL software package (version 1.5.6) was employed for docking purpose [27, 28]. Both the allele (HLA-C 07∗02) and ligand (epitope) files were firstly converted into PDBQT format to use them for the docking study. The grid/space box center was set at −15.059, −3.063, and −26.955 Å in the *x*-, *y*-, and *z*-axes, respectively, to allow the epitope to bind to the binding groove of the HLA-C 07∗02. The size was set at 20, 40, and 40 Å in the *x*, *y*, and *z* dimensions, respectively. For predicting binding of 15-mer epitopes in the binding groove of class II allele (HLA-DR1 or DRB1∗01:01), the grid/space box center was set at 9.292, 26.935, and 40.729 Å in the *x*-, *y*-, and *z*-axes, respectively (after converting DRB1∗01:01 and epitope files into PDBQT format). The size was set at 58, 22, and 38 Å in the *x*, *y*, and *z* dimensions, respectively.

All the analyses were done at 1.00- Å spacing. The exhaustiveness parameter (that influences the thoroughness of global search algorithm) was kept at 8.00, while the number of outputs was set at 10. These parameters were performed in AutoDOCK tool. The docking was conducted using AutoDOCK Vina program based on the above-mentioned parameters. All the output PDBQT files were converted in PDB format using OpenBabel (version 2.3.1) and visualized in PyMOL molecular Graphics system. The best output was selected on the basis of higher binding energy.

#### 2.7.2. Control

The 3D structure of MHC class I H-2Kb molecule complexed with octapeptide PKB1 (“KVITFIDL”) was retrieved from Protein Data Bank Database (ID: IKJ3) and visualized using PyMOL Graphics. The octapeptide was excluded before applying the structure of H-2Kb for comparing the validated data obtained for predicted structure of HLA-C 07∗02.

Also, to assess HLA-C 07∗02-epitope docking results, octapeptide PKB1 (“KVITFIDL”) was used as the control. This peptide was docked with HLAs, HLA-C 07∗02, and H-2Kb. The test epitope(s) and the control peptide were docked by setting similar parameters for each trial and successful binding of this peptide to these HLAs was demonstrated. Finally, H-2Kb - KVITFIDL docking result was used as control to compare with the test docking results of HLA-C 07∗02 complexed with selected epitopes. A comparative analysis of the best binding energy (Kcal/mol) and the arrangement of the test and control epitope at the binding groove of MHC allele HLA-C 07∗02 was also performed.

Crystal structure of HLA-DRB1 complexed with an endogenous peptide (“GSDWRFLRGYHQYA”) was retrieved from protein databank (PDB ID: 1AQD). The peptide was excluded from the structure and its binding to the binding groove of HLA-DRB1 was used as the control model to compare the binding models of the predicted epitopes “SEWISIVPNFILVRN” and “VFYQASFSWDTMIKF” using AutoDOCK Vina.

## 3. Results

### 3.1. Retrieval of Protein Sequences and Identification and Selection of Conserved Sequences

A total of 18 sequences of G and F proteins of different isolates of NiV have been retrieved. CLUSTALW programme in MEGA software generated several conserved sequences with varying lengths. A total of 15 and 8 conserved sequences were found in G and F proteins of NiV, respectively. Conserved sequences generated in this method have been presented in Table 1.

### 3.2. Antigenicity and Transmembrane Properties of the Conserved Sequences

Analysis revealed that 9 and 5 conserved sequences, respectively, from G and F proteins met the criteria of default threshold level, ≥0.4, in VaxiJen (Table 1). On the other hand, transmembrane topology showed that of all VaxiJen passed conserved sequences, 5 sequences each from G (out of 9) and F (out of 5) proteins fulfilled the criteria of exomembrane characteristics (Table 1).

### 3.3. Identification of T Cell Epitopes

T cell epitopes are processed peptides which could be recognized by the T cell receptors presented through Class I/Class II molecules on antigen presenting cells. VaxiJen analyses were used for the identification and reevaluation of T cell epitopes because some T cell epitopes tend to lose the antigenicity when reanalyzed. So, to increase the confidence level of prediction about the epitopes presented as a good T cell based epitope, these were cross-checked with the VaxiJen scores.

#### 3.3.1. MHC Class I Epitope Identification and Selection from Conserved Sequences

NetCTL prediction tool covering all supertypes created a total of 146 and 151 nonamers from the conserved sequences of G and F proteins, respectively. Analysis in SMM based IEDB MHC I prediction tool retrieved 59 T cell epitopes (peptides from G protein) that interacted with 29 possible MHC I alleles with the IC_50_ value <250 nM. Using the same IC_50_ value in case of conserved sequences of F protein, 37 different MHC class I molecules showed binding interaction with 150 T cell epitopes ranging from two to as many as ten MHC class I alleles. Next step analyses were proceeded with the peptides that showed interaction with ≥5 MHC class I alleles and had VaxiJen score ≥0.5. A total of 26 nonamers from F protein and 12 nonamers from G protein fulfilled these criteria which have been presented in Table 2. Further observation and evaluation revealed that of all predicted sequences “IGFCLITKR” epitope derived from fusion protein had the highest VaxiJen score (2.4587). Further, “ISCPNPLPF” and “SLIDTSSTI” (VaxiJen scores: 0.9212 and 0.509, resp.) from G protein and “TVNPSLISM” (VaxiJen score: 0.7251) from F protein interacted with as many as 8 MHC class I alleles.

#### 3.3.2. MHC Class II Epitope Identification and Selection from Conserved Sequences

While a study reported that a binding affinity (IC_50_) threshold of 500 nM identifies peptide binders recognized by T cells and this threshold can be used to select peptides [29], we kept binding affinity within 250 nM to get better confidence level in predicting epitopes for MHC Class II alleles. This generated 15 amino acid residues containing 96 and 273 peptides, respectively, from the conserved sequences of G and F proteins that showed interaction with many different and/or common MHC II alleles with an IC_50_ value ranging from 3 to 250 nM. A good epitope should also interact with as many as MHC alleles. Thus, among the total peptides, it was found that only 10 peptides from G protein while 69 peptides from F protein showed interaction with ≥5 MHC Class II alleles. To find out the most probable peptide-based epitopes with better confidence level, selected peptides were further tested using VaxiJen score and peptides having score of ≥0.5 were annotated. Sixteen peptides from F protein and 9 peptides from G protein could be considered as the most potential epitopes for MHC Class II alleles. Among all the most probable epitopes “IPANIGLLGSKISQS” from glycoprotein and “SNIEIGFCLITKRSV” from fusion proteins had the highest VaxiJen scores of 1.4106 and 1.8516, respectively. The most probable epitopic candidates interacting with several MHC class II alleles along with their VaxiJen scores have been shown in Table 3.

### 3.4. Population Coverage and Epitope Conservancy

Over a thousand different human MHC (HLA) alleles are known and different HLA types are expressed at different frequencies in different ethnicities. Identified epitopes that bind to several MHC alleles would be considered as the best probable epitope only if their combined frequency in a population show good coverage by approaching 100% or close to 100%.

**Figure 2:**
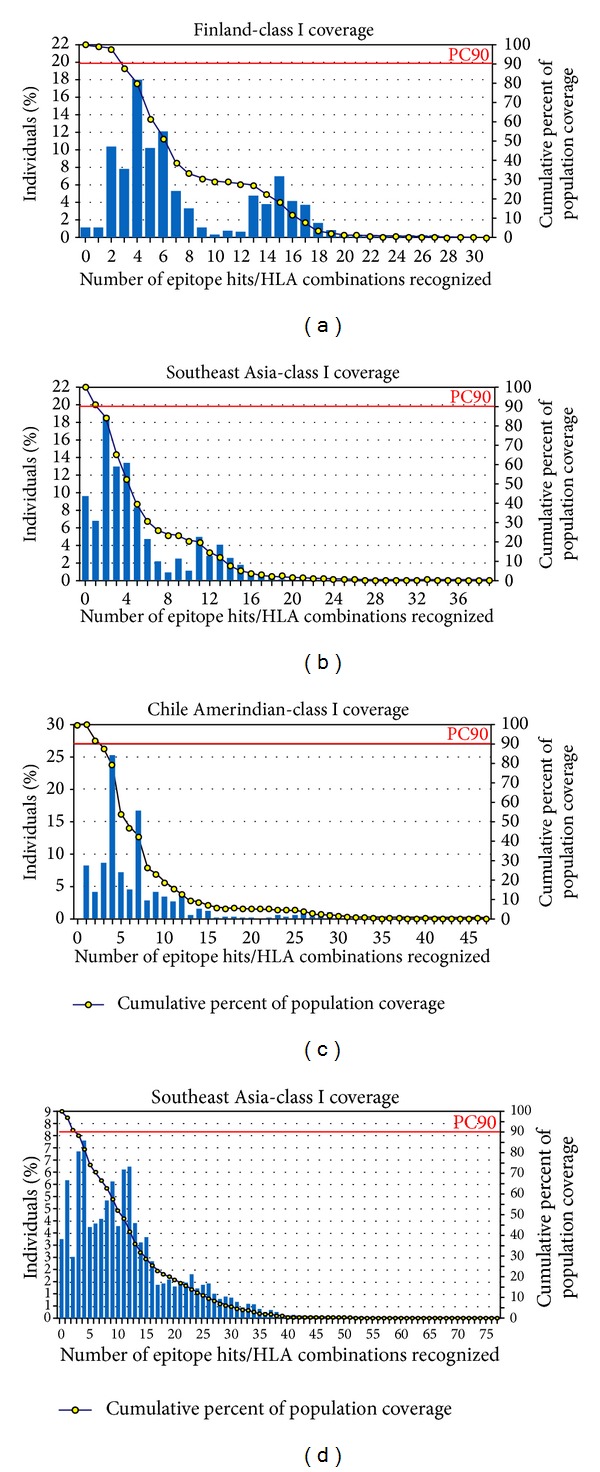
Population coverage by MHC Class I restricted epitopes predicted from G and F proteins of NiV. In case of epitopes from G protein: (a) shows maximum coverage by the population of Finland and (b) represents coverage by the overall population of South-East Asia. In case of epitopes from F protein: (c) displays the highest coverage by the population of Cheli Amerindian and (d) depicts coverage by the overall population of South-East Asia.

Elicitation of the immune response of the >90% (average value) world population could be covered by the most potential nine epitopes predicted from glycoprotein G. Maximum coverage 98.84% (Figure 2(a)) was found in the population of Finland and Finland Caucasoid followed by 97.39%, 96.70%, and 96.65% in the population of Mexico Amerindian, Philippines and Philippines Austronesian, and United States Polynesian, respectively. Population of Southeast Asia and South Asia showed maximum coverage of 90.44% and 85.19%, respectively. On the other hand, nonamers derived from F protein showed an average coverage value of 97.72% among the world population. The highest coverage, 100% (Figure 2(c)), was obtained in Chile Amerindian population followed by 99.98%, 99.96%, 99.71, and 99.61% coverage by the population of Peru and Peru Amerindian, Mexico Amerindian, United States Amerindian, and United States Polynesian, respectively. Figures 2(b) and 2(d) represent class I coverage by the overall population of Southeast Asia. Supplementary File 1, in Supplementary Material available online at http://dx.doi.org/10.1155/2014/402492, represents descending order of percentages of class I coverage by the all populations present in the database.

Conservation analyses of epitopes (http://tools.immuneepitope.org/tools/conservancy/iedb_input) with all G and F protein sequences from different strains of NiV demonstrated that the predicted epitopes were conserved among their respective G and F protein sequences.

### 3.5. Prediction and Selection of B Cell Epitopes

According to the criteria set for the prediction of B cell epitopes, Table 4 demonstrated the epitopes predicted from F and G proteins using ABCpred server and on the basis of VaxiJen scores. F protein generated 24 predicted peptides (scores: 0.54–0.75) while G protein generated 18 predicted peptides (scores: 0.52–0.85) which could be considered as the probable B cell epitopes. Among all these epitopes “VSNMSQCTEI” and “GEQTLLMIDN” from F protein showed the highest score of 0.75 while epitope “SQSTASINEN” from G protein had the highest score of 0.85 (not shown in the table). With regard to antigenic scores obtained from VaxiJen, “ISVTCQCQTT” and “LKNKIWCISL” sequences derived, respectively, from F and G proteins had the highest antigenic scores of 1.0871 and 2.3553, respectively (Table 4). Further, out of these sequences, only “EWISIVPNFI” decamer from F protein as well as “EISDQRLSIG” and “SLIDTSSTIT” from G protein showed sequence similarity with the superimposed T cell nonameric epitopes.

### 3.6. Description of the Three-Dimensional Structure of HLA-C 07∗02

**Figure 3:**
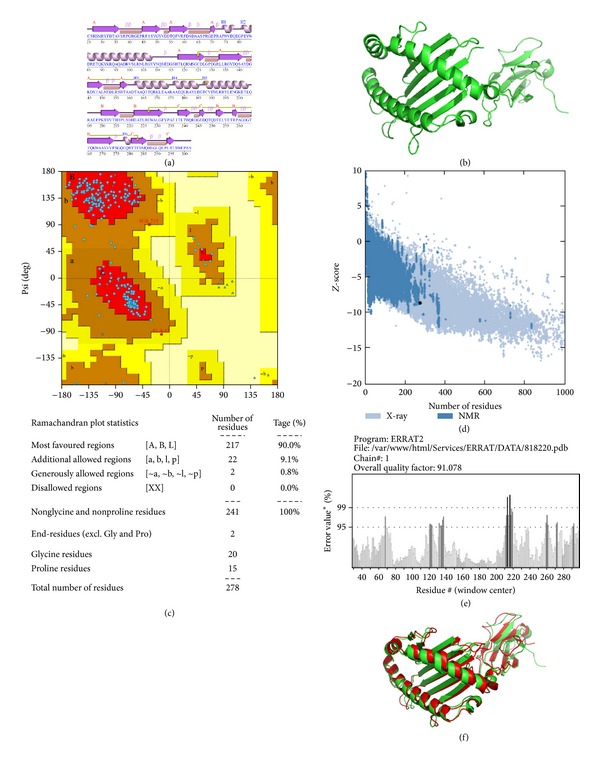
Quality of the predicted 3D structure of HLA-C∗07:02. The structure was predicted using phyre2 protein modeling software. (a), (b), (c), (d), and (e), respectively, indicate the secondary structure of the HLA-C∗07:02 MHC class I molecule including labeled helices H1, H2 with beta and gamma turns and hairpin; predicted 3-dimensional structure of the HLA-C∗07:02 protein; Ramachandran plot along with statistics; Z-score for quality of the 3D structure and (f) represents superimposed alpha helical structure of H-2Kb on predicted HLA-C∗07:02.

Figures 3(a) and 3(b) represent the secondary and the predicted 3D structure of* Homo sapiens* MHC class I HLA-C 07∗02 allele, respectively. Predicted structure was evaluated using Z-score, ERRAT, and Ramachandran plots to verify its quality and reliability. To assess stereochemical quality of the structure, PROCHECK tool was used. This tool using Ramachandran plot showed that >90% of residues are in the favorable region (Figure 3(c)) that ultimately assured the quality of the protein structure. PROSA web tool provided Z-score (signify overall model quality) that determined whether the structure is within the range of scores found in native proteins of comparable size. The Z-score of the protein was −8.73 (Figure 3(d)). Results from ERRAT showed that overall model quality of the predicted structure was 91.078% (Figure 3(e)) that was almost similar to that of H-2Kb molecule (93.704%) that reassured the reliability of the model. The Z-scores, Ramachandran plot, and ERRAT results confirmed the quality of the homology model of the HLA-C 07∗02. Further, the predicted three-dimensional structure of HLA-C 07∗02 was superimposed on the alpha chain of H-2Kb and it was found that the two structures were completely overlaid on each other (Figure 3(f)).

### 3.7. Molecular Docking of HLA-Epitope Interaction

**Figure 4:**
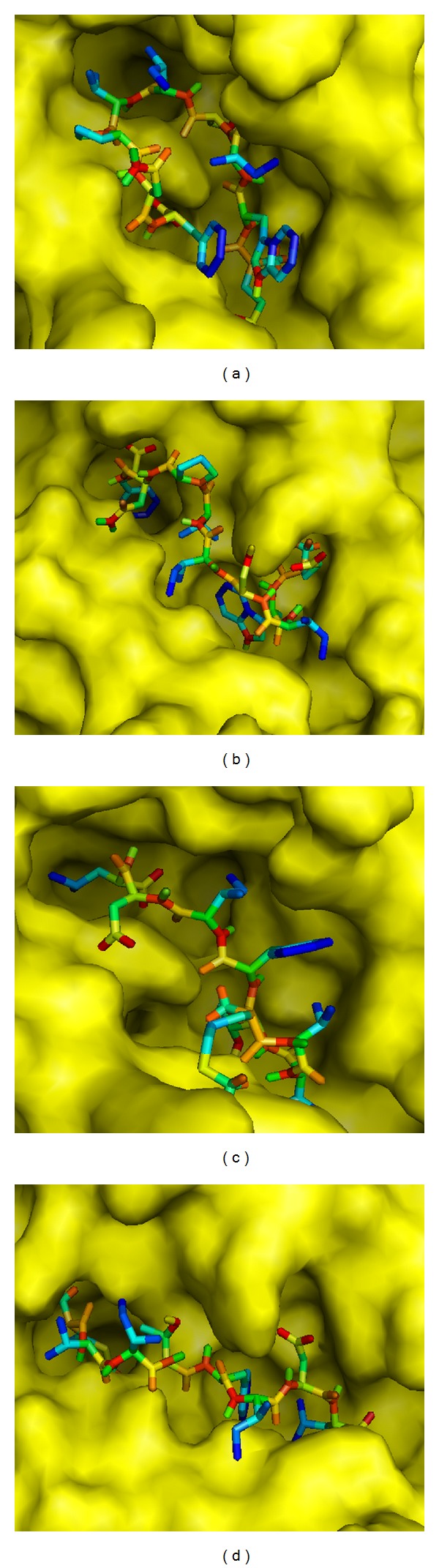
Docking to predict the binding of predicted and control epitopes to MHC class I molecule, HLA-C∗07:02 and H-2Kb. Binding of “EWISIVPNF” to the binding grooves (a) of the predicted structure of HLA-C∗07:02 (binding energy: −6.9 Kcal/mol) and (b) of the 3D structure of H-2Kb (binding energy: −7.7 Kcal/mol); (c) binding of control peptide (PKB1, KVITFIDL) to the predicted 3D structure of HLA-C∗07:02 (−6.7 Kcal/mol) and (d) H-2Kb (−7.3 Kcal/mol).

**Figure 5:**
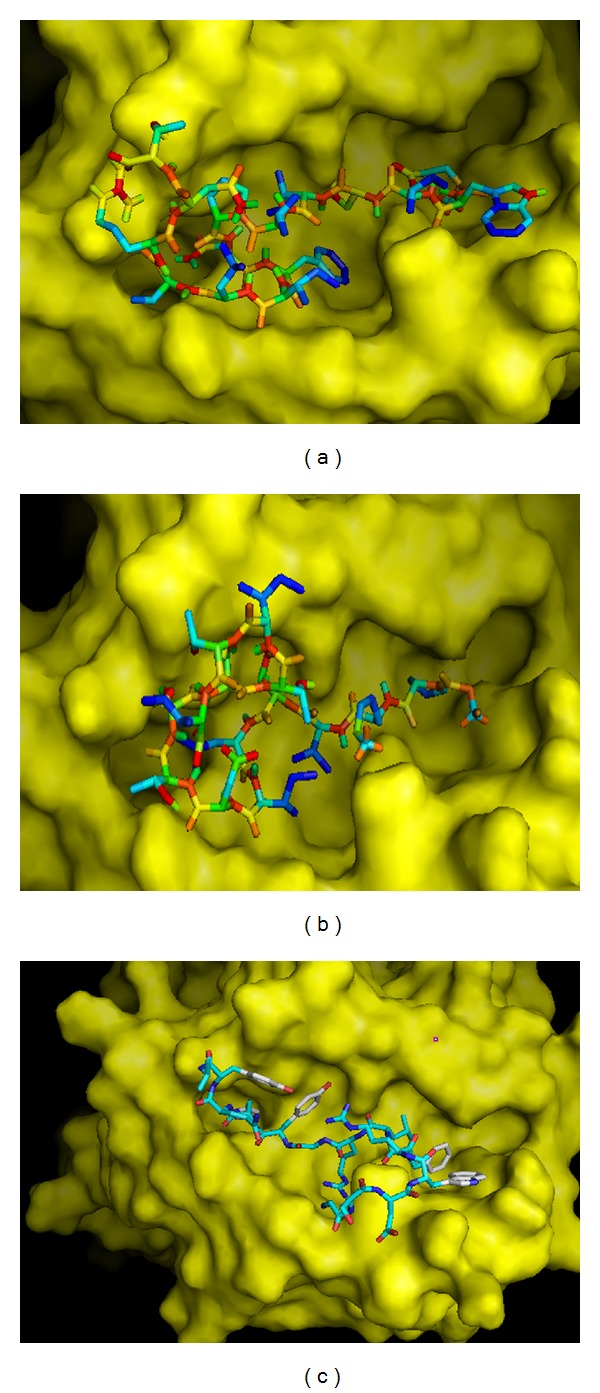
Docking to predict the binding of predicted and control epitopes to MHC class II molecule, HLA-DRB1∗01:01. Binding of containing predicted epitopes to the binding groove of class II MHC allele HLA-DRB1∗01:01. (a) “EWISIVPNFILVRNT” (–6.6 Kcal/mol); (b) “GPKVSLIDTSSTITI” (−4.6 Kcal/mol); (c) endogenous peptide A2 (GSDWRFLRGYHQYA) (−6.2 Kcal/mol).

Using AutoDock Vina, binding models of predicted epitopes to their respective HLA molecules (both class I and class II) were generated (Figures 4 and 5). In case of class I HLA-C07∗02, epitope “EWISIVPNF” bound to the binding groove with the binding energy −6.9 Kcal/mol (Figure 4(a)). Also, this epitope bound to the binding groove of H-2Kb with the binding energies of −7.7 Kcal/mol (Figure 4(b)). Control peptide “KVITFIDL” bound to the grooves of HLA-C07∗02 and H-2Kb with the binding energies −6.7 Kcal/mol and −7.3 Kcal/mol, respectively (Figures 4(c) and 4(d)).

The binding affinities (reflected by the lower binding energies) for the “EWISIVPNFILVRNT” and “GPKVSLIDTSSTITI” to DRB1∗01:01 were estimated to be −6.6 Kcal/mol and −4.6 Kcal/mol, respectively, while for the control peptide “GSDWRFLRGYHQYA”, it was −6.2 Kcal/mol. The binding models generated from Molecular docking using AutoDOCK Vina have been presented in Figure 5.

## 4. Discussion

The world is now the habitat of more than seven billion people. With the advent of medical technology, new kinds of diseases are also emerging along with new viruses. Developing world, in particular, is more affected by these sorts of diseases. Diseases which have earlier been recognized as zoonotic are now spreading from human to human. However, medical science has always tried to cope with the problems with the pace of replicating disease. Nipah virus infection is one of the reasons of fatalities in humans and livestock in countries like Australia, Bangladesh, India, Malaysia, and Singapore. Nipah outbreaks have resulted in acute respiratory distress syndrome and encephalitis, person-to-person transmission with fatality rates of 40–75% in humans [1–4]. Although a number of NiV vaccine studies have been reported, till date, there is no vaccines or drugs licensed for human use. In this study, we made an attempt to design epitopes which could be tested for their efficacy in eliciting immunity through humoral and cell mediated immune responses. The glycoprotein G and fusion protein F facilitate the attachment and fusion of NiV with host cell membranes [30]. For these two vital involvements at the gateway, these proteins were targeted for designing most potential epitopes using* in silico* computational methods after retrieving sequences from the databases. Conserved sequences which were used for the analyses have been presented in Table 1.

Most antigens and vaccines trigger not only B cell response but also T cell response. Vaccine induces production of antibodies that are synthesized by B cells and mediates effector functions by binding specifically to a toxin or a pathogen [31]. However, over time, humoral response from memory B cells can easily be overcome by surge of antigens while cell mediated immunity often elicits lasting immunity [32, 33]. Cytotoxic CD8^+^T lymphocytes (CTL) restrict the spread of infectious agents by recognizing and killing infected cells or secreting specific antiviral cytokines [34]. Thus, T cell epitope-based vaccination is a unique process of eliciting strong immune response against infectious agents, for example, viruses [35]. Peptide antigens are generally 8–10 amino acids long, with residues involved in either MHC or TCR binding or both. Specificity for class I binding is largely conferred by two or three dominant anchor residues [36], while antigen specificity of MHC-peptide complex recognition is generally determined by the few side chains of the peptide antigen that are solvent-exposed (between one and three residues) and available for T-cell receptor (TCR) contacts. This paradigm strengthens the basis of software algorithms that predict 8-9-mer class I epitopes from protein sequences. In the current study, peptide lengths were set at 9 before making software based class I T cell epitope prediction using immune epitope database (IEDB). Predicted nonameric epitopes showed good population coverage. On the other hand, in case of MHC Class II epitopes, it was found that alleles for only HLA DRB1 are present in the database for calculating population coverage, while other alleles like HLA DRB4 or HLA DRBA5 are absent. As a result, population coverage for class II alleles was not considered in this study. However, the data from Table 3 demonstrated that all the 15-mer epitopes showed interaction with one of the most common HLA alleles, HLA-DRB1∗01:01. Epitope conservancy analysis in IEDB revealed that all the predicted epitopes showed very good conservancy with 100% protein sequence match. This is to mention here that the IEDB [37, 38] is conceivably the most wide-ranging database of experimentally characterized B cell and T cell epitopes. It provides users with access to several epitope-related analysis and prediction tools that allows retrieving both intrinsic biochemical and extrinsic context dependent information about epitopes [37]. This makes it possible to easily assemble customized datasets [39]. Furthermore, meta-analyses of pathogens of interest [40–42] were accomplished by several researchers using IEDB that further boosts up its utility in the analysis and prediction of epitopes.

Initial observation of the data demonstrated that among all the predicted epitopes “IGFCLITKR,” a F protein derived peptide, has the highest VaxiJen score 2.4587 while “TVNPSLISM” derived from F protein as well as “ISCPNPLPF” and “SLIDTSSTI” from G protein showed interaction with the highest numbers of MHC class I alleles (as many as eight alleles, Table 2). Thus, either of these peptides could be regarded as the most probable epitope. Moreover, according to VaxiJen scores, the “SNIEIGFCLITKRSV” and “IPANIGLLGSKISQS” could be the most probable candidates (Table 3). On the other hand, with regard to the coverage of MHC alleles, the most probable peptides are “DTLYFPAVGFLVRTE”, “GDTLYFPAVGFLVRT” and “TLYFPAVGFLVRTEF” from G protein as they interact with seven different class II alleles while the most probable peptide “PNFILVRNTLISNIE” from F protein as it interacts with ten different class II alleles.

However, further investigation of the data revealed that “EWISIVPNF” and “ISIVPNFIL” class I epitopes from F protein have been recognized as the core sequences of four Class II overlapping epitopes that include “SEWISIVPNFILVRN,” “DNSEWISIVPNFILV,” “EWISIVPNFILVRNT,” and “NDNSEWISIVPNFIL” sequences, while epitopes “FILVRNTLI,” “VGILHYEKL,” “IGFCLITKR,” and “YEKLSKIG” were found as the core sequences of “NFILVRNTLISNIEI,” “SVGILHYEKLSKIGL,” “EIGFCLITKRSVICN,” and “SVGILHYEKLSKIGL” epitopes, respectively. On the other hand, in case of G protein, “ITIPANIGL,” “VFYQASFSW,” and “SLIDTSSTI” share sequence with MHC Class II epitopes “ITIPANIGLLGSKIS,” “VFYQASFSWDTMIKF,” and “GPKVSLIDTSSTITI,” while “FPAVGFLVR” and “YFPAVGFLV” as well as “TLYFPAVGF” and “VGFLVRTEF” Class I epitopes share common sequences of Class II epitopes “DTLYFPAVGFLVRTE” and “TLYFPAVGFLVRTEF,” respectively. All these MHC class II epitopes were found to interact with one of the most common HLA alleles, HLA-DRB1∗01:01. These data have been presented in Tables 2 and 3. Moreover, the overlapping prospective class II epitopes with a single core nonamer presented in Table 3 were not redundant because each variety of class II HLA molecule has a unique peptide binding pocket and prefers distinct amino acids at certain positions of the peptide. This peptide preference is mainly determined by the primary and auxiliary anchor residues, where one specific or a closely related amino acid is required for efficient peptide binding [43, 44]. In addition, peptide residues immediately flanking the core region have been indicated to make contact with the MHC molecule outside of the binding groove and to contribute to MHC-peptide interaction [45]. This has been reflected by the fact that the overlapping sequences (Table 3) even having the same core nonamer bind to varying numbers of class II MHC alleles with different binding affinities. Recently, Yadav and Mishra (2013) reported that QTEGVSNLV and LMMTRLV epitopes from glycoprotein G of NiV had highest binding affinity for MHC Class I HLA-A∗01:01 and HLA-A∗02:01, respectively [46]. In their study, the sequence of glycoprotein G of NiV was retrieved from http://www.uniprot.org (Accession Number Q9IH62) after ignoring the sequences of glycoprotein G from other isolates from the NiV endemic regions. In the present study, all the available sequences of glycoprotein G and fusion protein F were retrieved from databases and multiple sequence alignment generated conserved sequences which were tested in VaxiJen to predict their probable antigenicity. This, we believe, would generate more acceptable epitope(s) that should be effective universally.

Along with the T-cell epitope, attention was also given to the B-cell epitope, which can induce primary and secondary humoral immunity. Out of the total B cell epitopes (25 from F protein and 18 from G protein), only 7 and 13 epitopes, respectively, from F and G proteins with VaxiJen score of ≥0.5 were selected for further analysis (Table 4). When T cell epitopes (nonamers from F and G proteins) were superimposed, only “EWISIVPNF” from F protein showed similarity with the linear B cell decameric epitope “EWISIVPNFI.” On the other hand, “SLIDTSSTI” and “FIEISDQRL” from G protein showed similarity with the decameric epitopes “SLIDTSSTIT” and “EISDQRLSIG” (7 amino acid overlap). The rationale behind such evaluation is due to the relay hypothesis [47] which hypothesized that superimposition of CTL epitopes with B cell epitope and T helper epitope would ensure good T cell response with specific T cell memory and will be beneficial for the formulation of the vaccine. In addition, among the T and B cell epitopes studied, 15-mer “EWISIVPNFILVRNT” epitope from F protein completely showed sequence similarity to two decameric B cell epitopes “EWISIVPNFI” and “VPNFILVRNT” along with the nonameric Class I T cell epitope “EWISIVPNF,” while other three 15 mers (“SEWISIVPNFILVRN,” “DNSEWISIVPNFILV,” and “NDNSEWISIVPNFIL”) showed partial similarity. Similarly, in case of epitopes from G protein, “ANIGLLGSKISQSTA” class II epitope had sequence similarity with B cell epitope “IGLLGSKISQ,” while only “GPKVSLIDTSSTITI” had similarity with both the B cell epitope “SLIDTSSTIT” and superimposed class I epitope “SLIDTSSTI.” Considering all these observations, nonamers “EWISIVPNF” and “SLIDTSSTI” as well as 15 mers “EWISIVPNFILVRNT” and “GPKVSLIDTSSTITI,” respectively, from the fusion and glycoproteins of NiV were considered as the most probable candidates for vaccine development.

The Class I T cell epitopes “EWISIVPNF” and “SLIDTSSTI” have been found to interact with HLA-C∗03:03, HLA-C∗12:03, and HLA-C∗14:02 alleles with varying affinities except HLA-C∗07:02, HLA-A∗23:01, HLA-A∗02:01, HLA-B∗ 15:02, HLA-A∗02:06, HLA-A∗32:01, and HLA-B15∗01 alleles. When the frequencies of these alleles were analyzed among the world population in http://www.allelefrequencies.net, the highest frequency was observed in case of HLA-C∗07:02 in Australian, Asian (Indian, Chinese, Japanese, and Pakistanis), Europeans, and South Americans compared to the frequencies of other alleles. Thus, for docking, 3D structure of HLA-C∗07:02 was used with the predicted structure of “EWISIVPNF.” As “SLIDTSSTI” epitope did not show any interaction with HLA-C∗07:02 allele, this nonamer was not used for docking. The binding energies of predicted epitope “EWISIVPNF” for its binding to the binding grooves of HLA-C∗07:02 were −6.9 Kcal/mol (Figure 4(a)). On the other hand, this peptide could also bind to the binding groove of H-2Kb having almost similar affinity with the binding energies found to be −7.7 Kcal/mol (Figure 4(b)). Further, the binding energies of the control peptide (PKB1, KVITFIDL) to the binding grooves of HLA-C∗07:02 and H-2Kb were found to be almost close to those of the predicted epitopes, −6.7 and −7.3 Kcal/mol, respectively (Figures 4(c) and 4(d)). Due to lack of crystal structure of HLA-C∗07:02 along with foreign peptide, we could not show binding of control peptide with this allele for comparing our docking result. HLA-C alleles are considered to have apparently minor role in mediating antigen-specific T cell response along with its low expression on the cell surface. However, it has been observed that HIV-1 Nef has the ability to selectively downregulate HLA class I A and B molecules to minimize cytotoxic T lymphocyte scrutiny while maintaining HLA-C expression and this has led to an approval of the role of HLA-C as a T-cell restriction element, particularly in HIV-1 infection [48]. Besides, HLA-C alleles are often in strong linkage disequilibrium with HLA-B alleles, making it difficult to distinguish HLA-C from HLA-B-restricted responses. Sometimes HLA-C shares sequence homology with other classical human class I HLA-A and HLA-B molecules. Moreover, HLA-C epitopes have been mistakenly identified as restricted by HLA-A or HLA-B (e.g., some B14 epitopes in HIV-1 p24 are now thought to be Cw8-restricted, Los Alamos Immunology Database [49]). Thus, it is indeed important to verify the rationale behind the identification of HLA-C restricted probable epitopes using animal models or* in vivo* studies. On the other hand, “EWISIVPNFILVRNT” and “GPKVSLIDTSSTITI” 15 mers were docked to test their interaction with the binding cleft of one of the most important and prevalent class II MHC molecules HLA-DRB1∗01:01. The binding energies of these two predicted epitopes were –6.6 and −4.6 kcal/mol (Figures 5(a) and 5(b)). This binding energy was compared with the binding energy of endogenous 14mer epitope (“GSDWRFLRGYHQYA”) to HLA-DRB1∗01:01 and found to be the same as the predicted epitope (−6.2 kcal/mol, Figure 5(c)). Almost similar binding energy of the simulations of “EWISIVPNFILVRNT” and the control peptide “GSDWRFLRGYHQYA” indicates the satisfactory accuracy of the predicted epitope though “GPKVSLIDTSSTITI” peptide showed relatively higher binding affinity.

## 5. Conclusion

Experimental approaches for predicting epitopes eliciting both humoral and T cell immunity are time-consuming, costly, and not applicable to the large scale screening. Computer modeling methods can help to minimize the number of experiments by scanning systematically best candidate peptides with higher population coverage and interaction of these peptides with as many as HLA alleles can ultimately bring a momentum in vaccine development. Our proposed predicted epitopes “EWISIVPNF” and “SLIDTSSTI” (that overlap/superimpose on class II and B cell decameric epitopes) showed good MHC class I coverage by the world population even by the population from Southeast Asia where Nipah virus infection has been reported (Figures 2(b) and 2(d)) along with 100% epitope conservancy. However, aptness of these peptides as probable vaccine would be accepted upon successful experiments using model animals.

## Supplementary Material

Supplementary Table 1 (a) represents the coverage of the HLA alleles by different population shown by the epitopes predicted from F protein while (b) indicates overage of the HLA alleles by different population shown by the epitopes predicted from G protein of NiV.

## Figures and Tables

**(a):** 

F-protein	Position of the selected sequences	Prediction of transmembrane helix using TMHMM server	VaxiJen value
ISECSVGILHYEKLSKIGLVKG	19–41	Outside	0.67

TRKYKIKSNPLTKDIVIKMI	43–61	Inside	0.17

NVSNMSQCTEIYKNNTHDLVGDVRLAGVIMAGSVMENYKTRLNGILT PIKGALGVAIGIATAAQITAGVALYEAMKNADNINKLKSSIESTNEAVV KLQETAEKTVYVLTALQDYINTNLVPTIDKISCKQTELSLDLALSKY	64–112	Outside	0.44
	113–135	TM helix	
	136–206	Inside	

SDLLFVFGPNLQDPVSNSMTIQAISQAFGGNYETLLRTLGYA	208–249	Outside	0.21

FDDLLESDSITGQIIYVDLS	253–272	Outside	0.39

YYIIVRVYFPILTEIQQAYIQELLPVSFNNDNSEWISIVPNFILVRNTLISNI EIGFCLITKRSVICNQDYATP	274–347	Outside	0.72

TNNMRECLTGSTEKCPRELVVSSHVPRFALSNGVLFANCISVTCQCQT TGRAISQSGEQTLLMIDNTTCPTAVLGNVIISLGKYLGSVNYNSEGIAIG PPVFTDKVDISSQ	349–459	Outside	0.41

SSMNQSLQQSKDYIKEAQRLLDTVNPSLISMLSMIILYVLSIASLCIGLIT FISFIIVEKKRNTYSRLEDRRVRPTSSGDLYYIGT	461–494	Outside	0.7251
	495–517	TM helix	
	518–546	Inside	

**(b):** 

G protein	Position of the selected sequences	Prediction of transmembrane helix using TMHMM server	VaxiJen value
PSKVIKSYYGTMDIKKINEGLLDSKILSAFNTVIALLGSIVIIVMNIMIIQN YTRSTDNQA	21–47	Outside	0.38
	48–67	TM helix	
	68–81	Inside	

IQQQIKGLADKIGTEIGPKVSLIDTSSTITIPANIGLLGSKISQSTASINENV NEKCKFTLPPLKIHECNISCPNPLPFREY	89–171	Outside	0.62

PQTEGVSNLVGLP	173–185	Outside	0.2138

NICLQKTSNQILKPKLISYTLPVVGQSGTCITDPLLAMDEGYFAYSHLE	187–235	Inside	0.58

IGSCSRGVSKQRIIGVGEVLDRGDEVPSLFMTNVW	237–271	Inside	0.50

NPNTVYHCSAVYNNEFYYVLCAVS	275–298	Inside	0.28

LNSTYWSGSLMMTRLAVKPK	305–315	Inside	0.78

YDKVMPYGPSGIKQGDTLYFPAVGFLVRTEF	345–375	Outside	0.84

CQYSKPENCRLSMGIRPNSHYILRSGLLKYNLSD	387–421	Inside	0.27

YDSLGQPVFYQASFSWDTMIKFGDV	445–469	Outside	0.61

CWEGVYNDAFLIDRINWISAGVFLDSNQTAENPVFTVFKDNE	503–544	Outside	0.33

LYRAQLASEDTNAQKTITNCFLLKNKIWCISLVEIYDTGDNVIRPKLFA VKIPEQCT	546–602	Outside	0.59

VFIEISDQRLSIGSPSK	427–443	Outside	1.1465

TVNPLVVNWR	471–480	Inside	1.6557

NTVISRPGQSQCPRFN	482–497	Inside	−0.0237

**(a):** 

Epitopes from fusion protein	Interacting MHC Class I alleles	Position	VaxiJen score
EGIAIGPPV	HLA C∗1203, HLA C∗0303, HLA A∗6802, HLA C∗1402, HLA A∗0206	441–449	0.6844

EWISIVPNF	HLA C∗0303, HLA C∗1203, HLA C∗1402, HLA A∗2301, HLA C∗0702	307–315	0.72

FILVRNTLI	HLA C∗0303, HLA C∗1203, HLA C∗0701, HLA A∗0206, HLA A∗0201, HLA C∗0602	315–323	0.72

IIVRVYFPI	HLA C∗1203, HLA C∗0303, HLA A∗0206, HLA A∗6802, HLA A∗0201	276–284	0.72

ISIVPNFIL	HLA C∗0303, HLA B∗1502, HLA C∗1203, HLA B∗5801, HLA C∗1502	309–317	0.72

LLDTVNPSL	HLA C∗0501, HLA C∗1203, HLA A∗0201, HLA C∗1402, HLA B∗1502, HLA A∗0206	480–488	0.7251

TVNPSLISM	HLA C∗1203, HLA C∗0501, HLA C∗0303, HLA B∗3501, HLA C∗0702, HLA C∗1402, HLA B∗1502, HLA C∗0701	483–491	0.7251

VGILHYEKL	HLA C∗0303, HLA C∗1203, HLA B∗1502, HLA C∗0702, HLA C∗1402	25–33	0.67

VYFPILTEI	HLA C∗1402, HLA C∗1203, HLA A∗2301, HLA C∗0303, HLA C∗0602, HLA C∗0702, HLA C∗0701	280–288	0.72

AQITAGVAL	HLA C∗0303, HLA B∗3901, HLA A∗0206, HLA B∗1501, HLA B∗1502, HLA C∗1203, HLA B∗4001	126–134	0.813

EGIAIGPPV	HLA C∗1203, HLA C∗0303, HLA A∗6802, HLA C∗1402, HLA A∗0206	441–449	0.6844

IGFCLITKR	HLA C∗0303, HLA C∗1203, HLA A∗3101, HLA A∗6801, HLA C∗1402	328–336	2.4587

ISFIIVEKK	HLA C∗1203, HLA A∗6801, HLA A∗1101, HLA C∗1502, HLA C∗0303, HLA C∗1402	513–521	2.2805

ISMLSMIIL	HLA B∗1502, HLA C∗1203, HLA C∗1402, HLA C∗0303, HLA C∗1502	489–497	0.5006

ITFISFIIV	HLA C∗1203, HLA A∗6802, HLA A∗0206, HLA C∗1402, HLA A∗3001	510–518	0.914

LSLDLALSK	HLA C∗1502, HLA A∗3001, HLA A∗1101, HLA C∗1203, HLA C∗0303, HLA C∗1402	197–205	0.9847

ILTEIQQAY	HLA C∗1502, HLA C∗1203, HLA A∗1101, HLA C∗0303, HLA C∗1402, HLA A∗3001	284–292	0.721

MLSMIILYV	HLA-A∗02:01, HLA-A∗02:06, HLA-A∗68:02, HLA- C∗12:03, HLA-C∗14:02	491–499	0.6397

NTYSRLEDR	HLA A∗6801, HLA C∗0303, HLA C∗1203, HLA C∗1402, HLA C∗0701, HLA C∗1502, HLA A∗3101	523–531	1.5575

PTSSGDLYY	HLA A∗2902, HLA C∗1203, HLA C∗0303, HLA A∗0101, HLA B∗1502, HLA C∗1402, HLA C∗0501	535–543	0.5252

TAAQITAGV	HLA A∗6802, HLA C∗1203, HLA C∗0303, HLA C∗0501, HLA C∗1502	124–132	0.7373

TELSLDLAL	HLA C∗0303, HLA B∗1502, HLA B∗4001, HLA B∗4002, HLA C∗1203, HLA B∗3901, HLA C∗0702	195–203	0.9593

VRPTSSGDL	HLA B∗1502, HLA C∗0602, HLA C∗1402, HLA C∗1203, HLA C∗0701, HLA C∗0702	533–541	1.0043

YEKLSKIGL	HLA B∗1502, HLA C∗1203, HLA C∗0303, HLA B∗4001, HLA B∗4002	30–38	0.67

YIKEAQRLL	HLA C∗1203, HLA B∗1502, HLA C∗0701, HLA C∗0602, HLA C∗0303, HLA C∗0702	473–481	0.7251

YYIIVRVYF	HLA A∗2301, HLA C∗1402, HLA A∗2402, HLA C∗0702, HLA C∗1203, HLA C∗0303, HLA B∗1502	274–282	0.72

**(b):** 

Epitopes from glycoprotein	Interacting MHC Class I alleles	Position	VaxiJen score
FIEISDQRL	HLA B∗1502, HLA C∗0303, HLA C∗1203, HLA C∗0501, HLA C∗0702, HLA C∗1502	428	1.5463

FPAVGFLVR	HLA C∗0303, HLA C∗1203, HLA A∗6801, HLA B∗1502, HLA B∗3501	364–372	1.053

ISCPNPLPF	HLA C∗0303, HLA B∗5801, HLA C∗1203, HLA B∗1501, HLA B∗1502, HLA C∗1402, HLA C∗1502, HLA B∗3501	164–172	0.921

ITIPANIGL	HLA C∗0303, HLA C∗1203, HLA A∗6802, HLA B∗1502, HLA A∗0206, HLA C∗1502, HLA C∗1402	118–126	1.2697

TEIGPKVSL	HLA C∗0303, HLA B∗4001, HLA C∗1203, HLA B∗1502, HLA B∗4002, HLA B∗3901	103–111	1.337

TLYFPAVGF	HLA C∗0303, HLA C∗1402, HLA B∗1502, HLA C∗1203, HLA A∗3201	361–369	0.9600

VFYQASFSW	HLA C∗1203, HLA C∗1402, HLA C∗0303, HLA B∗5801, HLA A∗2301	452–460	1.2161

VGFLVRTEF	HLA C∗1203, HLA C∗0702, HLA C∗1402, HLA B∗1502, HLA C∗0303	367–375	1.443

YFPAVGFLV	HLA C∗1203, HLA A∗0206, HLA C∗1402, HLA A∗6802, HLA A∗0201	363–371	0.834

MPYGPSGIK	HLA C∗0303, HLA C∗1203, HLA B∗1502, HLA A∗0301, HLA C∗1402, HLA A∗1101	349–357	1.243

RLSIGSPSK	HLA C∗0303, HLA C∗1203, HLA A∗0301, HLA A∗3001, HLA C∗1402, HLA A∗1101	435–443	1.134

SLIDTSSTI	HLA C∗0303, HLA C∗1203, HLA A∗0201, HLA B∗1502, HLA C∗1402, HLA A∗0206, HLA A∗3201, HLA B∗1501	110–118	0.509

**(a):** 

Epitopes from fusion protein	Interacting class II MHC alleles	Antigenic scores	
FILVRNTLISNIEIG	HLA DRB1∗0701, HLA DRB1∗0101, HLA DRB1∗0405, HLA DRB5∗0101, HLA DRB1∗0404, HLA DRB1∗1101, HLA DRB1∗1302, HLA DRB1∗0401	1.109	315

EIGFCLITKRSVICN	HLA-DRB1∗11:01 HLA-DRB1∗07:01 HLA-DRB1∗01:01 HLA-DRB1∗04 HLA- DRB1∗09:01	1.4646	327

IEIGFCLITKRSVIC	HLA-DRB1∗11:01 HLA-DRB1∗07:01 HLA-DRB1∗01:01 HLA-DRB1∗04 HLA- DRB1∗09:01	1.7736	326

NIEIGFCLITKRSVI	HLA-DRB1∗11:01 HLA-DRB1∗07:01 HLA-DRB1∗01:01 HLA-DRB1∗04 HLA- DRB1∗09:01	1.5682	325

SNIEIGFCLITKRSV	HLA-DRB1∗11:01 HLA-DRB1∗07:01 HLA-DRB1∗01:01 HLA-DRB1∗04 HLA- DRB1∗09:01	1.8516	324

SVGILHYEKLSKIGL	HLA-DRB1∗11:01 HLA-DRB1∗01:01 HLA-DRB4∗01:01 HLA-DRB1∗04 HLA- DPA1∗03:01/DPB1∗04:02	1.3695	24

VGILHYEKLSKIGLV	HLA-DRB1∗11:01 HLA-DRB1∗01:01 HLA-DRB4∗01:01 HLA-DPA1∗03:01/DPB1∗04:02 HLA-DRB5∗01:01	1.2192	25

DNSEWISIVPNFILV	HLA DRB1∗0701, HLA DRB1∗0101, HLA DRB5∗0101, HLA DRB1∗0405, HLA DRB1∗1501, HLA DRB1∗1302, HLA DRB1∗0404, HLA DRB1∗09, HLA DPA1∗0201/DPB1∗0101	0.938	304

EWISIVPNFILVRNT	HLA DRB1∗0701, HLA DRB1∗1501, HLA DRB1∗0101, HLA DRB5∗0101, HLA DRB1∗0404, HLA DRB1∗0405, HLA DRB1∗1302, HLA DPA1∗0201/DPB1∗0101	0.781	307

IISLGKYLGSVNYNS	HLA DRB1∗0401, HLA DRB1∗0101, HLA DRB1∗0405, HLA DRB1∗0404, HLA DQA1∗0501/DQB1∗0301, HLA DRB1∗1101	0.633	426

NDNSEWISIVPNFIL	HLA DRB1∗0701, HLA DRB1∗0101, HLA DRB5∗0101, HLA DRB1∗0405, HLA DRB1∗1501, HLA DRB1∗1302, HLA DRB1∗0404, HLA DRB1∗09	0.58	303

NFILVRNTLISNIEI	HLA DRB1∗0701, HLA DRB1∗0101, HLA DRB1∗0405, HLA DRB5∗0101, HLA DRB1∗0404, HLA DRB1∗1101, HLA DRB1∗1302, HLA DRB1∗0401, HLA DRB1∗1501	0.74	314

PNFILVRNTLISNIE	HLA DRB1∗0701, HLA DRB1∗0101, HLA DRB1∗0405, HLA DRB5∗0101, HLA DRB1∗0404, HLA DRB1∗1101, HLA DRB1∗1302, HLA DRB1∗0401, HLA DRB1∗1501, HLA DRB4∗0101	0.612	313

SEWISIVPNFILVRN	HLA DRB1∗0701, HLA DRB1∗0101, HLA DRB5∗0101, HLA DRB1∗1501, HLA DRB1∗0405, HLA DRB1∗1302, HLA DRB1∗0404, HLA DRB1∗09, HLA DPA1∗0201/DPB1∗0101	0.728	306

WISIVPNFILVRNTL	HLA DRB1∗0701, HLA DRB1∗0101, HLA DRB5∗0101, HLA DRB1∗1501, HLA DRB1∗0405, HLA DRB1∗0404, HLA DRB1∗1302, HLA DPA1∗0201/DPB1∗0101	0.681	308

GILHYEKLSKIGLVK	HLA-DRB1∗11:01 HLA-DRB1∗01:01 HLA-DRB4∗01:01 HLA-DPA1∗03:01/DPB1∗04:02 HLA-DRB1∗09:01	0.6020	26

**(b):** 

Epitopes from glycoprotein	Interacting MHC class II alleles	VaxiJen scores	Position
ANIGLLGSKISQSTA	HLA DRB1∗0101, HLA DQA1∗0501/DQB1∗0301, HLA DRB1∗0404, HLA DRB1∗1501, HLA DRB1∗1101	1.1268	122–136

DTLYFPAVGFLVRTE	HLA DRB1∗0101, HLA DPA1∗0201/DPB1∗0101, HLA DPA1∗0103/DPB1∗0201, HLA DPA1∗01/DPB1∗0401, HLA-DPA1∗0301/DPB1∗0402, HLA DRB1∗0701, HLA DQA1∗0501/DQB1∗0301	0.9628	360–374

GDTLYFPAVGFLVRT	HLA DRB1∗0101, HLA DPA1∗0201/DPB1∗0101, HLA DPA1∗0103/DPB1∗0201, HLA DPA1∗01/DPB1∗0401, HLA DPA1∗0301/DPB1∗0402, HLA DRB1∗0701, HLA DQA1∗0501/DQB1∗0301	0.6663	359–373

GPKVSLIDTSSTITI	HLA DRB1∗0701, HLA DRB1∗1302, HLA DRB1∗0101, HLA DRB1∗0404, HLA DRB1∗0401	0.8609	106–120

IPANIGLLGSKISQS	HLA DRB1∗0101, HLA DQA1∗0501/DQB1∗0301, HLA DRB1∗0404, HLA DRB1∗1501, HLA DRB1∗1101	1.4106	120–134

ITIPANIGLLGSKIS	HLA DRB1∗0101, HLA DRB1∗0404, HLA DRB1∗1501, HLA DRB1∗1101, HLA DQA1∗0501/DQB1∗0301	1.2611	118–132

PANIGLLGSKISQST	HLA DRB1∗0101, HLA DQA1∗0501/DQB1∗0301, HLA DRB1∗0404, HLA DRB1∗1501, HLA DRB1∗1101	1.1782	121–135

TLYFPAVGFLVRTEF	HLA DRB1∗0101, HLA DPA1∗0201/DPB1∗0101, HLA DPA1∗01/DPB1∗0401, HLA DPA1∗0103/DPB1∗0201, HLA DPA1∗0301/DPB1∗0402, HLA DRB1∗0701, HLA DQA1∗0501/DQB1∗0301	1.1201	361–375

VFYQASFSWDTMIKF	HLA DRB3∗0101, HLA DRB1∗0701, HLA DPA1∗0103/DPB1∗0201, HLA DPA1∗0201/DPB1∗0101, HLA DRB1∗0101	0.507	452–466

**Table 4:** Predicted linear B cell epitopes.

Epitopes from fusion protein	VaxiJen Scores	ABCpred Scores	Epitopes from glycoprotein	VaxiJen Scores	ABCpred Scores
AYIQELLPVS	0.5449	0.72	SQSTASINEN	0.5024	0.85
HDLVGDVRLA	0.7435	0.72	EISDQRLSIG	1.6593	0.78
VPNFILVRNT	0.8719	0.68	TITIPANIGL	1.109	0.75
ISVTCQCQTT	1.0871	0.65	IGPKVSLIDT	0.6982	0.72
EWISIVPNFI	0.7234	0.61	DKVMPYGPSG	0.7825	0.7
LPVSFNNDNS	0.7892	0.58	QLASEDTNAQ	0.6684	0.7
YKNNTHDLVG	0.6676	0.56	IGLLGSKISQ	1.2309	0.66
			IGTEIGPKVS	1.3442	0.65
			LKNKIWCISL	2.3553	0.62
			PYGPSGIKQG	1.2519	0.6
			SLIDTSSTIT	0.5981	0.58
			NCFLLKNKIW	1.0781	0.53
			ISLVEIYDTG	0.7707	0.52

Decameric epitopes having antigenic score ≥0.5 were only considered.

## References

[1] Wang L, Harcourt BH, Yu M (2001). Molecular biology of Hendra and Nipah viruses. *Microbes and Infection*.

[2] Chua KB, Goh KJ, Wong KT (1999). Fatal encephalitis due to Nipah virus among pig-farmers in Malaysia. *The Lancet*.

[3] Chadha MS, Comer JA, Lowe L (2006). Nipah virus-associated encephalitis outbreak, Siliguri, India. *Emerging Infectious Diseases*.

[4] Gurley ES, Montgomery JM, Hossain MJ (2007). Person-to-person transmission of Nipah virus in a Bangladeshi community. *Emerging Infectious Diseases*.

[5] Kai C, Yoneda M (2011). Henipavirus infections—an expanding zoonosis from fruit Bats. *Journal of Disaster Research*.

[6] Mire CE, Versteeg KM, Cross RW (2013). Single injection recombinant vesicular stomatitis virus vaccines protect ferrets against lethal Nipah virus disease. *Virology Journal*.

[7] Guillaume V, Contamin H, Loth P (2004). Nipah virus: vaccination and passive protection studies in a hamster model. *Journal of Virology*.

[8] Bossart KN, Rockx B, Feldmann F (2012). A Hendra virus G glycoprotein subunit vaccine protects African green monkeys from Nipah virus challenge. *Science Translational Medicine*.

[9] Weingartl HM, Berhane Y, Caswell JL (2006). Recombinant Nipah virus vaccines protect pigs against challenge. *Journal of Virology*.

[10] Yoneda M, Georges-Courbot M, Ikeda F (2013). Recombinant measles virus vaccine expressing the Nipah virus glycoprotein protects against lethal Nipah virus challenge. *PLoS ONE*.

[11] Cerdeño-Tárraga AM, Efstratiou A, Dover LG (2003). The complete genome sequence and analysis of *Corynebacterium diphtheriae* NCTC13129. *Nucleic Acids Research*.

[12] Larsen JE, Lund O, Nielsen M (2006). Improved method for predicting linear B-cell epitopes. *Immunome Research*.

[13] Kuhns JJ, Batalia MA, Shuqin Y, Collins EJ (1999). Poor binding of a HER-2/neu epitope (GP2) to HLA-A2.1 is due to a lack of interactions with the center of the peptide. *Journal of Biological Chemistry*.

[14] Watts C (1997). Capture and processing of exogenous antigens for presentation on MHC molecules. *Annual Review of Immunology*.

[15] Germain RN (1994). MHC-dependent antigen processing and peptide presentation: providing ligands for T lymphocyte activation. *Cell*.

[16] Lafuente EM, Reche PA (2009). Prediction of MHC-peptide binding: a systematic and comprehensive overview. *Current Pharmaceutical Design*.

[17] Doytchinova IA, Flower DR (2007). VaxiJen: a server for prediction of protective antigens, tumour antigens and subunit vaccines. *BMC Bioinformatics*.

[18] Krogh A, Larsson B, Von Heijne G, Sonnhammer ELL (2001). Predicting transmembrane protein topology with a hidden Markov model: application to complete genomes. *Journal of Molecular Biology*.

[19] Larsen MV, Lundegaard C, Lamberth K (2005). An integrative approach to CTL epitope prediction: a combined algorithm integrating MHC class I binding, TAP transport efficiency, and proteasomal cleavage predictions. *European Journal of Immunology*.

[20] Nielsen M, Lundegaard C, Lund O (2007). Prediction of MHC class II binding affinity using SMM-align, a novel stabilization matrix alignment method. *BMC Bioinformatics*.

[21] Thévenet P, Shen Y, Maupetit J, Guyon F, Derreumaux P, Tufféry P (2012). PEP-FOLD: an updated de novo structure prediction server for both linear and disulfide bonded cyclic peptides. *Nucleic Acids Research*.

[22] Maupetit J, Derreumaux P, Tuffery P (2009). PEP-FOLD: an online resource for *de novo* peptide structure prediction. *Nucleic Acids Research*.

[23] Kelley LA, Sternberg MJE (2009). Protein structure prediction on the Web: a case study using the Phyre server. *Nature Protocols*.

[24] Laskowski RA, MacArthur MW, Moss DS, Thornton JM (1993). PROCHECK—a program to check the stereochemical quality of protein structures. *Journal of Applied Crystallography*.

[25] Wiederstein M, Sippl MJ (2007). ProSA-web: interactive web service for the recognition of errors in three-dimensional structures of proteins. *Nucleic Acids Research*.

[26] Colovos C, Yeates TO (1993). Verification of protein structures: patterns of nonbonded atomic interactions. *Protein Science*.

[27] Morris GM, Huey R, Lindstrom W (2009). AutoDock4 and AutoDockTools4: automated docking with selective receptor flexibility. *Journal of Computational Chemistry*.

[28] Morris GM, Goodsell DS, Halliday RS (1998). Automated docking using a Lamarckian genetic algorithm and an empirical binding free energy function. *Journal of Computational Chemistry*.

[29] Sette A, Vitiello A, Reherman B (1994). The relationship between class I binding affinity and immunogenicity of potential cytotoxic T cell epitopes. *The Journal of Immunology*.

[30] Bossart KN, Wang L, Flora MN (2002). Membrane fusion tropism and heterotypic functional activities of the *Nipah virus* and *Hendra virus* envelope glycoproteins. *Journal of Virology*.

[31] Cooper NR, Nemerow GR (1984). The role of antibody and complement in the control of viral infections. *Journal of Investigative Dermatology*.

[32] Bacchetta R, Gregori S, Roncarolo M-G (2005). CD4^+^ regulatory T cells: mechanisms of induction and effector function. *Autoimmunity Reviews*.

[33] Igietseme JU, Eko FO, He Q, Black CM (2004). Antibody regulation of T-cell immunity: implications for vaccine strategies against intracellular pathogens. *Expert Review of Vaccines*.

[34] Garcia KC, Teyton L, Wilson IA (1999). Structural basis of T cell recognition. *Annual Review of Immunology*.

[35] Shrestha B, Diamond MS (2004). Role of CD8^+^ T cells in control of West Nile virus infection. *Journal of Virology*.

[36] Rammensee HG, Falk K, Rötzschke O (1993). Peptides naturally presented by MHC class I molecules. *Annual Review of Immunology*.

[37] Peters B, Sidney J, Bourne P (2005). The immune epitope database and analysis resource: from vision to blueprint. *PLoS Biology*.

[38] Zhang Q, Wang P, Kim Y (2008). Immune epitope database analysis resource (IEDB-AR). *Nucleic acids research*.

[39] Sollner J, Grohmann R, Rapberger R, Perco P, Lukas A, Mayer B (2008). Analysis and prediction of protective continuous B-cell epitopes on pathogen proteins. *Immunome Research*.

[40] Vaughan K, Blythe M, Greenbaum J (2009). Meta-analysis of immune epitope data for all Plasmodia: overview and applications for malarial immunobiology and vaccine-related issues. *Parasite Immunology*.

[41] Zarebski LM, Vaughan K, Sidney J (2008). Analysis of epitope information related to *Bacillus anthracis* and *Clostridium botulinum*. *Expert Review of Vaccines*.

[42] Blythe M, Zhang Q, Vaughan K (2007). An analysis of the epitope knowledge related to Mycobacteria. *Immunome Research*.

[43] Falk K, Rotzschke O, Stevanovic S, Jung G, Rammensee H-G (1991). Allele-specific motifs revealed by sequencing of self-peptides eluted from MHC molecules. *Nature*.

[44] Hunt DF, Henderson RA, Shabanowitz J (1992). Characterization of peptides bound to the class I MHC molecule HLA-A2.1 by mass spectrometry. *Science*.

[45] Godkin AJ, Smith KJ, Willis A (2001). Naturally processed HLA class II peptides reveal highly conserved immunogenic flanking region sequence preferences that reflect antigen processing rather than peptide-MHC interactions. *Journal of Immunology*.

[46] Yadav PK, Mishra M (2013). Computational epitope prediction and docking studies of glycoprotein-G in Nipah virus. *International Journal of Bioinformatics and Biological Science*.

[47] Lal G, Shaila MS, Nayak R (2006). Activated mouse T cells downregulate, process and present their surface TCR to cognate anti-idiotypic CD4+ T cells. *Immunology and Cell Biology*.

[48] Collins KL, Chen BK, Kalams SA, Walker BD, Baltimore D (1998). HIV-1 Nef protein protects infected primary cells against killing by cytotoxic T lymphocytes. *Nature*.

[49] Yusim K, Korber BTM, Christian B (2009). *HIV Molecular Immunology*.

