# Supplementary material for: Prediction of Epitope-Based Peptides for the Utility of Vaccine Development from Fusion and Glycoprotein of Nipah Virus Using In Silico Approach
Source: Adv Bioinformatics. 2014 Jul 24;2014:402492. doi: 10.1155/2014/402492 (PMC4131549; doi:10.1155/2014/402492)
Supplement: Supplementary file 1 — Supplementary Table 1 (a) represents the coverage of the HLA alleles by different population shown by the epitopes predicted from F protein while (b) indicates overage of the HLA alleles by different population shown by the epitopes predicted from G protein of NiV. [file 402492.f1.doc]

*For Fusion Protein(F):*

| Population / Area | Class I | | |
| --- | --- | --- | --- |
|  | **Coverage ^a^** | **Average hit ^b^** | **PC90 ^c^** |
| [World](http://tools.immuneepitope.org/tools/population/PopulationCalculationServlet#World) | 97.72% | 13.32 | 3.58 |
| [Chile Amerindian](http://tools.immuneepitope.org/tools/population/PopulationCalculationServlet#Chile%20Amerindian) | 100.00% | 6.83 | 2.46 |
| [Peru](http://tools.immuneepitope.org/tools/population/PopulationCalculationServlet#Peru) | 99.98% | 5.31 | 3.29 |
| [Peru Amerindian](http://tools.immuneepitope.org/tools/population/PopulationCalculationServlet#Peru%20Amerindian) | 99.98% | 4.87 | 3.24 |
| [Mexico Amerindian](http://tools.immuneepitope.org/tools/population/PopulationCalculationServlet#Mexico%20Amerindian) | 99.96% | 13.98 | 5.29 |
| [United States Amerindian](http://tools.immuneepitope.org/tools/population/PopulationCalculationServlet#United%20States%20Amerindian) | 99.71% | 10.46 | 3.29 |
| [United States Polynesian](http://tools.immuneepitope.org/tools/population/PopulationCalculationServlet#United%20States%20Polynesian) | 99.61% | 19.43 | 4.17 |
| [England Caucasoid](http://tools.immuneepitope.org/tools/population/PopulationCalculationServlet#England%20Caucasoid) | 99.53% | 15.57 | 5.78 |
| [Ireland Northern](http://tools.immuneepitope.org/tools/population/PopulationCalculationServlet#Ireland%20Northern) | 99.52% | 14.04 | 5.65 |
| [Ireland Northern Caucasoid](http://tools.immuneepitope.org/tools/population/PopulationCalculationServlet#Ireland%20Northern%20Caucasoid) | 99.52% | 14.04 | 5.65 |
| [Ireland South](http://tools.immuneepitope.org/tools/population/PopulationCalculationServlet#Ireland%20South) | 99.51% | 14.04 | 5.64 |
| [Ireland South Caucasoid](http://tools.immuneepitope.org/tools/population/PopulationCalculationServlet#Ireland%20South%20Caucasoid) | 99.51% | 14.04 | 5.64 |
| [Bulgaria Other](http://tools.immuneepitope.org/tools/population/PopulationCalculationServlet#Bulgaria%20Other) | 99.41% | 11.36 | 3.91 |
| [Papua New Guinea](http://tools.immuneepitope.org/tools/population/PopulationCalculationServlet#Papua%20New%20Guinea) | 99.41% | 12.47 | 3.07 |
| [Papua New Guinea Melanesian](http://tools.immuneepitope.org/tools/population/PopulationCalculationServlet#Papua%20New%20Guinea%20Melanesian) | 99.41% | 12.47 | 3.07 |
| [Australia Caucasoid](http://tools.immuneepitope.org/tools/population/PopulationCalculationServlet#Australia%20Caucasoid) | 99.38% | 12.85 | 5.07 |
| [Philippines](http://tools.immuneepitope.org/tools/population/PopulationCalculationServlet#Philippines) | 99.31% | 15.59 | 4.52 |
| [Philippines Austronesian](http://tools.immuneepitope.org/tools/population/PopulationCalculationServlet#Philippines%20Austronesian) | 99.31% | 15.59 | 4.52 |
| [Finland](http://tools.immuneepitope.org/tools/population/PopulationCalculationServlet#Finland) | 99.27% | 16.21 | 5.21 |
| [Finland Caucasoid](http://tools.immuneepitope.org/tools/population/PopulationCalculationServlet#Finland%20Caucasoid) | 99.27% | 16.21 | 5.21 |
| [Germany](http://tools.immuneepitope.org/tools/population/PopulationCalculationServlet#Germany) | 99.20% | 15.07 | 5.05 |
| [Germany Caucasoid](http://tools.immuneepitope.org/tools/population/PopulationCalculationServlet#Germany%20Caucasoid) | 99.20% | 15.07 | 5.05 |
| [United States Caucasoid](http://tools.immuneepitope.org/tools/population/PopulationCalculationServlet#United%20States%20Caucasoid) | 99.18% | 14.65 | 4.87 |
| [France](http://tools.immuneepitope.org/tools/population/PopulationCalculationServlet#France) | 98.99% | 14.71 | 4.74 |
| [France Caucasoid](http://tools.immuneepitope.org/tools/population/PopulationCalculationServlet#France%20Caucasoid) | 98.99% | 14.71 | 4.74 |
| [Poland](http://tools.immuneepitope.org/tools/population/PopulationCalculationServlet#Poland) | 98.94% | 15.89 | 4.74 |
| [Poland Caucasoid](http://tools.immuneepitope.org/tools/population/PopulationCalculationServlet#Poland%20Caucasoid) | 98.94% | 15.89 | 4.74 |
| [Russia Other](http://tools.immuneepitope.org/tools/population/PopulationCalculationServlet#Russia%20Other) | 98.84% | 13.98 | 3.67 |
| [England](http://tools.immuneepitope.org/tools/population/PopulationCalculationServlet#England) | 98.78% | 12.32 | 4.04 |
| [Russia Siberian](http://tools.immuneepitope.org/tools/population/PopulationCalculationServlet#Russia%20Siberian) | 98.78% | 13.7 | 3.36 |
| [Mexico](http://tools.immuneepitope.org/tools/population/PopulationCalculationServlet#Mexico) | 98.78% | 12.72 | 4.13 |
| [Europe](http://tools.immuneepitope.org/tools/population/PopulationCalculationServlet#Europe) | 98.63% | 14.68 | 4.42 |
| [Taiwan](http://tools.immuneepitope.org/tools/population/PopulationCalculationServlet#Taiwan) | 98.57% | 12.4 | 3.28 |
| [Taiwan Oriental](http://tools.immuneepitope.org/tools/population/PopulationCalculationServlet#Taiwan%20Oriental) | 98.57% | 12.4 | 3.28 |
| [Saudi Arabia](http://tools.immuneepitope.org/tools/population/PopulationCalculationServlet#Saudi%20Arabia) | 98.53% | 12.67 | 4.75 |
| [Saudi Arabia Arab](http://tools.immuneepitope.org/tools/population/PopulationCalculationServlet#Saudi%20Arabia%20Arab) | 98.53% | 12.67 | 4.75 |
| [United States Mestizo](http://tools.immuneepitope.org/tools/population/PopulationCalculationServlet#United%20States%20Mestizo) | 98.49% | 13.01 | 3.61 |
| [South Africa Other](http://tools.immuneepitope.org/tools/population/PopulationCalculationServlet#South%20Africa%20Other) | 98.46% | 11.96 | 3.61 |
| [New Caledonia](http://tools.immuneepitope.org/tools/population/PopulationCalculationServlet#New%20Caledonia) | 98.45% | 7.89 | 1.36 |
| [New Caledonia Melanesian](http://tools.immuneepitope.org/tools/population/PopulationCalculationServlet#New%20Caledonia%20Melanesian) | 98.45% | 7.89 | 1.36 |
| [Morocco Caucasoid](http://tools.immuneepitope.org/tools/population/PopulationCalculationServlet#Morocco%20Caucasoid) | 98.42% | 11.12 | 4.25 |
| [Japan](http://tools.immuneepitope.org/tools/population/PopulationCalculationServlet#Japan) | 98.38% | 14.46 | 2.47 |
| [Japan Oriental](http://tools.immuneepitope.org/tools/population/PopulationCalculationServlet#Japan%20Oriental) | 98.38% | 14.46 | 2.47 |
| [Korea; South](http://tools.immuneepitope.org/tools/population/PopulationCalculationServlet#Korea;%20South) | 98.35% | 14.91 | 3.2 |
| [Korea; South Oriental](http://tools.immuneepitope.org/tools/population/PopulationCalculationServlet#Korea;%20South%20Oriental) | 98.35% | 14.91 | 3.2 |
| [East Asia](http://tools.immuneepitope.org/tools/population/PopulationCalculationServlet#East%20Asia) | 98.29% | 14.41 | 2.63 |
| [Russia](http://tools.immuneepitope.org/tools/population/PopulationCalculationServlet#Russia) | 98.29% | 13.79 | 3.28 |
| [American Samoa](http://tools.immuneepitope.org/tools/population/PopulationCalculationServlet#American%20Samoa) | 98.27% | 9.64 | 2.44 |
| [American Samoa Polynesian](http://tools.immuneepitope.org/tools/population/PopulationCalculationServlet#American%20Samoa%20Polynesian) | 98.27% | 9.64 | 2.44 |
| [Italy](http://tools.immuneepitope.org/tools/population/PopulationCalculationServlet#Italy) | 98.21% | 15.77 | 4.41 |
| [Italy Caucasoid](http://tools.immuneepitope.org/tools/population/PopulationCalculationServlet#Italy%20Caucasoid) | 98.21% | 15.77 | 4.41 |
| [Bulgaria](http://tools.immuneepitope.org/tools/population/PopulationCalculationServlet#Bulgaria) | 98.13% | 11.41 | 4.02 |
| [Morocco](http://tools.immuneepitope.org/tools/population/PopulationCalculationServlet#Morocco) | 98.08% | 10.17 | 4.04 |
| [Venezuela Amerindian](http://tools.immuneepitope.org/tools/population/PopulationCalculationServlet#Venezuela%20Amerindian) | 98.06% | 10.6 | 3.45 |
| [United States Hispanic](http://tools.immuneepitope.org/tools/population/PopulationCalculationServlet#United%20States%20Hispanic) | 97.97% | 12.43 | 3.37 |
| [North America](http://tools.immuneepitope.org/tools/population/PopulationCalculationServlet#North%20America) | 97.96% | 11.88 | 3.44 |
| [United States](http://tools.immuneepitope.org/tools/population/PopulationCalculationServlet#United%20States) | 97.96% | 11.94 | 3.46 |
| [Venezuela](http://tools.immuneepitope.org/tools/population/PopulationCalculationServlet#Venezuela) | 97.96% | 10.57 | 3.45 |
| [United States Asian](http://tools.immuneepitope.org/tools/population/PopulationCalculationServlet#United%20States%20Asian) | 97.82% | 14.03 | 3.26 |
| [Morocco Arab](http://tools.immuneepitope.org/tools/population/PopulationCalculationServlet#Morocco%20Arab) | 97.64% | 8.89 | 3.55 |
| [Georgia Caucasoid](http://tools.immuneepitope.org/tools/population/PopulationCalculationServlet#Georgia%20Caucasoid) | 97.62% | 15.24 | 3.98 |
| [Oceania](http://tools.immuneepitope.org/tools/population/PopulationCalculationServlet#Oceania) | 97.42% | 10.72 | 1.82 |
| [Australia](http://tools.immuneepitope.org/tools/population/PopulationCalculationServlet#Australia) | 97.26% | 12.9 | 2.81 |
| [South Africa](http://tools.immuneepitope.org/tools/population/PopulationCalculationServlet#South%20Africa) | 97.24% | 11.95 | 3.76 |
| [South Africa](http://tools.immuneepitope.org/tools/population/PopulationCalculationServlet#South%20Africa) | 97.24% | 11.95 | 3.76 |
| [Sudan](http://tools.immuneepitope.org/tools/population/PopulationCalculationServlet#Sudan) | 97.22% | 13.91 | 4.26 |
| [Tunisia](http://tools.immuneepitope.org/tools/population/PopulationCalculationServlet#Tunisia) | 97.21% | 11.4 | 3.32 |
| [Tunisia Arab](http://tools.immuneepitope.org/tools/population/PopulationCalculationServlet#Tunisia%20Arab) | 97.21% | 11.4 | 3.32 |
| [Argentina](http://tools.immuneepitope.org/tools/population/PopulationCalculationServlet#Argentina) | 97.04% | 4.62 | 2.23 |
| [Argentina Amerindian](http://tools.immuneepitope.org/tools/population/PopulationCalculationServlet#Argentina%20Amerindian) | 97.04% | 4.62 | 2.23 |
| [Czech Republic](http://tools.immuneepitope.org/tools/population/PopulationCalculationServlet#Czech%20Republic) | 96.98% | 10.23 | 4.06 |
| [Czech Republic Caucasoid](http://tools.immuneepitope.org/tools/population/PopulationCalculationServlet#Czech%20Republic%20Caucasoid) | 96.98% | 10.23 | 4.06 |
| [Brazil Mixed](http://tools.immuneepitope.org/tools/population/PopulationCalculationServlet#Brazil%20Mixed) | 96.80% | 12.44 | 3.02 |
| [Brazil Amerindian](http://tools.immuneepitope.org/tools/population/PopulationCalculationServlet#Brazil%20Amerindian) | 96.72% | 10.49 | 2.74 |
| [Southeast Asia](http://tools.immuneepitope.org/tools/population/PopulationCalculationServlet#Southeast%20Asia) | 96.71% | 11.74 | 2.4 |
| [Georgia](http://tools.immuneepitope.org/tools/population/PopulationCalculationServlet#Georgia) | 96.53% | 13.81 | 3.08 |
| [Singapore Oriental](http://tools.immuneepitope.org/tools/population/PopulationCalculationServlet#Singapore%20Oriental) | 96.04% | 11.44 | 2.75 |
| [Australia Australian Aborigines](http://tools.immuneepitope.org/tools/population/PopulationCalculationServlet#Australia%20Australian%20Aborigines) | 95.97% | 12.13 | 1.92 |
| [Portugal](http://tools.immuneepitope.org/tools/population/PopulationCalculationServlet#Portugal) | 95.95% | 10.51 | 1.84 |
| [Portugal Caucasoid](http://tools.immuneepitope.org/tools/population/PopulationCalculationServlet#Portugal%20Caucasoid) | 95.95% | 10.51 | 1.84 |
| [Northeast Asia](http://tools.immuneepitope.org/tools/population/PopulationCalculationServlet#Northeast%20Asia) | 95.89% | 13.01 | 2.6 |
| [Chile](http://tools.immuneepitope.org/tools/population/PopulationCalculationServlet#Chile) | 95.74% | 10.11 | 1.75 |
| [Chile](http://tools.immuneepitope.org/tools/population/PopulationCalculationServlet#Chile) | 95.74% | 10.11 | 1.75 |
| [Brazil](http://tools.immuneepitope.org/tools/population/PopulationCalculationServlet#Brazil) | 95.67% | 11.6 | 2.35 |
| [China](http://tools.immuneepitope.org/tools/population/PopulationCalculationServlet#China) | 95.60% | 12.79 | 2.37 |
| [China Oriental](http://tools.immuneepitope.org/tools/population/PopulationCalculationServlet#China%20Oriental) | 95.60% | 12.79 | 2.37 |
| [North Africa](http://tools.immuneepitope.org/tools/population/PopulationCalculationServlet#North%20Africa) | 95.58% | 10.76 | 2.81 |
| [Pakistan Mixed](http://tools.immuneepitope.org/tools/population/PopulationCalculationServlet#Pakistan%20Mixed) | 95.48% | 9.74 | 1.74 |
| [Austria](http://tools.immuneepitope.org/tools/population/PopulationCalculationServlet#Austria) | 95.25% | 4.82 | 1.44 |
| [Austria Caucasoid](http://tools.immuneepitope.org/tools/population/PopulationCalculationServlet#Austria%20Caucasoid) | 95.25% | 4.82 | 1.44 |
| [Sweden](http://tools.immuneepitope.org/tools/population/PopulationCalculationServlet#Sweden) | 95.12% | 4.33 | 1.43 |
| [Sweden Caucasoid](http://tools.immuneepitope.org/tools/population/PopulationCalculationServlet#Sweden%20Caucasoid) | 95.12% | 4.33 | 1.43 |
| [Vietnam](http://tools.immuneepitope.org/tools/population/PopulationCalculationServlet#Vietnam) | 95.11% | 13.12 | 1.92 |
| [Vietnam Oriental](http://tools.immuneepitope.org/tools/population/PopulationCalculationServlet#Vietnam%20Oriental) | 95.11% | 13.12 | 1.92 |
| [Mexico Mestizo](http://tools.immuneepitope.org/tools/population/PopulationCalculationServlet#Mexico%20Mestizo) | 94.93% | 4.91 | 1.44 |
| [Iran](http://tools.immuneepitope.org/tools/population/PopulationCalculationServlet#Iran) | 94.58% | 12.43 | 3.19 |
| [Iran Persian](http://tools.immuneepitope.org/tools/population/PopulationCalculationServlet#Iran%20Persian) | 94.58% | 12.43 | 3.19 |
| [Thailand](http://tools.immuneepitope.org/tools/population/PopulationCalculationServlet#Thailand) | 94.33% | 11.86 | 2.75 |
| [Thailand Oriental](http://tools.immuneepitope.org/tools/population/PopulationCalculationServlet#Thailand%20Oriental) | 94.33% | 11.86 | 2.75 |
| [Singapore](http://tools.immuneepitope.org/tools/population/PopulationCalculationServlet#Singapore) | 94.25% | 10.57 | 1.68 |
| [Israel Arab](http://tools.immuneepitope.org/tools/population/PopulationCalculationServlet#Israel%20Arab) | 94.16% | 10.71 | 1.93 |
| [Romania](http://tools.immuneepitope.org/tools/population/PopulationCalculationServlet#Romania) | 94.15% | 3.86 | 1.29 |
| [Romania Caucasoid](http://tools.immuneepitope.org/tools/population/PopulationCalculationServlet#Romania%20Caucasoid) | 94.15% | 3.86 | 1.29 |
| [Pakistan](http://tools.immuneepitope.org/tools/population/PopulationCalculationServlet#Pakistan) | 93.81% | 10.76 | 1.68 |
| [Hong Kong](http://tools.immuneepitope.org/tools/population/PopulationCalculationServlet#Hong%20Kong) | 93.80% | 6.62 | 1.46 |
| [Hong Kong Oriental](http://tools.immuneepitope.org/tools/population/PopulationCalculationServlet#Hong%20Kong%20Oriental) | 93.80% | 6.62 | 1.46 |
| [South Asia](http://tools.immuneepitope.org/tools/population/PopulationCalculationServlet#South%20Asia) | 93.69% | 11.01 | 1.59 |
| [Spain](http://tools.immuneepitope.org/tools/population/PopulationCalculationServlet#Spain) | 93.69% | 13.88 | 4.05 |
| [Spain Caucasoid](http://tools.immuneepitope.org/tools/population/PopulationCalculationServlet#Spain%20Caucasoid) | 93.69% | 13.88 | 4.05 |
| [Uganda](http://tools.immuneepitope.org/tools/population/PopulationCalculationServlet#Uganda) | 93.55% | 9.3 | 1.68 |
| [Uganda Black](http://tools.immuneepitope.org/tools/population/PopulationCalculationServlet#Uganda%20Black) | 93.55% | 9.3 | 1.68 |
| [Croatia](http://tools.immuneepitope.org/tools/population/PopulationCalculationServlet#Croatia) | 93.42% | 3.73 | 1.22 |
| [Croatia Caucasoid](http://tools.immuneepitope.org/tools/population/PopulationCalculationServlet#Croatia%20Caucasoid) | 93.42% | 3.73 | 1.22 |
| [Georgia Kurd](http://tools.immuneepitope.org/tools/population/PopulationCalculationServlet#Georgia%20Kurd) | 93.26% | 10.45 | 1.49 |
| [United States Black](http://tools.immuneepitope.org/tools/population/PopulationCalculationServlet#United%20States%20Black) | 93.04% | 8.82 | 1.59 |
| [Sudan Mixed](http://tools.immuneepitope.org/tools/population/PopulationCalculationServlet#Sudan%20Mixed) | 92.92% | 12.86 | 1.57 |
| [Pakistan Asian](http://tools.immuneepitope.org/tools/population/PopulationCalculationServlet#Pakistan%20Asian) | 92.87% | 11.19 | 1.61 |
| [South America](http://tools.immuneepitope.org/tools/population/PopulationCalculationServlet#South%20America) | 92.69% | 9.17 | 1.39 |
| [Mali](http://tools.immuneepitope.org/tools/population/PopulationCalculationServlet#Mali) | 92.61% | 8.04 | 1.61 |
| [Mali Black](http://tools.immuneepitope.org/tools/population/PopulationCalculationServlet#Mali%20Black) | 92.61% | 8.04 | 1.61 |
| [Singapore Austronesian](http://tools.immuneepitope.org/tools/population/PopulationCalculationServlet#Singapore%20Austronesian) | 92.43% | 10.29 | 1.29 |
| [Belgium](http://tools.immuneepitope.org/tools/population/PopulationCalculationServlet#Belgium) | 92.30% | 3.75 | 1.15 |
| [Belgium Caucasoid](http://tools.immuneepitope.org/tools/population/PopulationCalculationServlet#Belgium%20Caucasoid) | 92.30% | 3.75 | 1.15 |
| [Kenya](http://tools.immuneepitope.org/tools/population/PopulationCalculationServlet#Kenya) | 92.14% | 7.52 | 1.34 |
| [Kenya Black](http://tools.immuneepitope.org/tools/population/PopulationCalculationServlet#Kenya%20Black) | 92.14% | 7.52 | 1.34 |
| [Bulgaria Caucasoid](http://tools.immuneepitope.org/tools/population/PopulationCalculationServlet#Bulgaria%20Caucasoid) | 91.85% | 3.75 | 1.14 |
| [Israel Jew](http://tools.immuneepitope.org/tools/population/PopulationCalculationServlet#Israel%20Jew) | 91.61% | 6.79 | 1.12 |
| [India](http://tools.immuneepitope.org/tools/population/PopulationCalculationServlet#India) | 91.52% | 10.2 | 1.21 |
| [India Asian](http://tools.immuneepitope.org/tools/population/PopulationCalculationServlet#India%20Asian) | 91.52% | 10.2 | 1.21 |
| [Brazil Caucasoid](http://tools.immuneepitope.org/tools/population/PopulationCalculationServlet#Brazil%20Caucasoid) | 91.43% | 3.71 | 1.1 |
| [Chile Mixed](http://tools.immuneepitope.org/tools/population/PopulationCalculationServlet#Chile%20Mixed) | 91.17% | 8.84 | 1.15 |
| [Mongolia](http://tools.immuneepitope.org/tools/population/PopulationCalculationServlet#Mongolia) | 90.95% | 3.32 | 1.04 |
| [Mongolia Oriental](http://tools.immuneepitope.org/tools/population/PopulationCalculationServlet#Mongolia%20Oriental) | 90.95% | 3.32 | 1.04 |
| [Southwest Asia](http://tools.immuneepitope.org/tools/population/PopulationCalculationServlet#Southwest%20Asia) | 90.76% | 9.16 | 1.12 |
| [Zimbabwe](http://tools.immuneepitope.org/tools/population/PopulationCalculationServlet#Zimbabwe) | 90.17% | 6.92 | 1.03 |
| [Zimbabwe Black](http://tools.immuneepitope.org/tools/population/PopulationCalculationServlet#Zimbabwe%20Black) | 90.17% | 6.92 | 1.03 |
| [East Africa](http://tools.immuneepitope.org/tools/population/PopulationCalculationServlet#East%20Africa) | 90.14% | 7.35 | 1.02 |
| [West Africa](http://tools.immuneepitope.org/tools/population/PopulationCalculationServlet#West%20Africa) | 89.71% | 6.79 | 0.97 |
| [Cuba Caucasoid](http://tools.immuneepitope.org/tools/population/PopulationCalculationServlet#Cuba%20Caucasoid) | 89.68% | 3.43 | 0.97 |
| [West Indies](http://tools.immuneepitope.org/tools/population/PopulationCalculationServlet#West%20Indies) | 89.50% | 3.6 | 0.95 |
| [Israel](http://tools.immuneepitope.org/tools/population/PopulationCalculationServlet#Israel) | 89.39% | 7.73 | 0.94 |
| [Cuba](http://tools.immuneepitope.org/tools/population/PopulationCalculationServlet#Cuba) | 89.06% | 3.55 | 0.91 |
| [Cameroon](http://tools.immuneepitope.org/tools/population/PopulationCalculationServlet#Cameroon) | 88.98% | 7.99 | 0.91 |
| [Cameroon Black](http://tools.immuneepitope.org/tools/population/PopulationCalculationServlet#Cameroon%20Black) | 88.98% | 7.99 | 0.91 |
| [Zambia](http://tools.immuneepitope.org/tools/population/PopulationCalculationServlet#Zambia) | 88.60% | 6.76 | 0.88 |
| [Zambia Black](http://tools.immuneepitope.org/tools/population/PopulationCalculationServlet#Zambia%20Black) | 88.60% | 6.76 | 0.88 |
| [Cuba Mulatto](http://tools.immuneepitope.org/tools/population/PopulationCalculationServlet#Cuba%20Mulatto) | 88.54% | 3.74 | 0.87 |
| [Senegal](http://tools.immuneepitope.org/tools/population/PopulationCalculationServlet#Senegal) | 87.81% | 6.04 | 0.82 |
| [Senegal Black](http://tools.immuneepitope.org/tools/population/PopulationCalculationServlet#Senegal%20Black) | 87.81% | 6.04 | 0.82 |
| [Cape Verde](http://tools.immuneepitope.org/tools/population/PopulationCalculationServlet#Cape%20Verde) | 87.61% | 3.41 | 0.81 |
| [Cape Verde Black](http://tools.immuneepitope.org/tools/population/PopulationCalculationServlet#Cape%20Verde%20Black) | 87.61% | 3.41 | 0.81 |
| [United Kingdom](http://tools.immuneepitope.org/tools/population/PopulationCalculationServlet#United%20Kingdom) | 87.26% | 10.04 | 3.14 |
| [United Kingdom Caucasoid](http://tools.immuneepitope.org/tools/population/PopulationCalculationServlet#United%20Kingdom%20Caucasoid) | 87.26% | 10.04 | 3.14 |
| [Central Africa](http://tools.immuneepitope.org/tools/population/PopulationCalculationServlet#Central%20Africa) | 86.91% | 7.95 | 0.76 |
| [Oman](http://tools.immuneepitope.org/tools/population/PopulationCalculationServlet#Oman) | 86.76% | 3.4 | 0.76 |
| [Oman Arab](http://tools.immuneepitope.org/tools/population/PopulationCalculationServlet#Oman%20Arab) | 86.76% | 3.4 | 0.76 |
| [Jordan](http://tools.immuneepitope.org/tools/population/PopulationCalculationServlet#Jordan) | 86.07% | 7.21 | 0.72 |
| [Jordan Arab](http://tools.immuneepitope.org/tools/population/PopulationCalculationServlet#Jordan%20Arab) | 86.07% | 7.21 | 0.72 |
| [Malaysia Oriental](http://tools.immuneepitope.org/tools/population/PopulationCalculationServlet#Malaysia%20Oriental) | 85.93% | 4.3 | 0.71 |
| [Guinea-Bissau](http://tools.immuneepitope.org/tools/population/PopulationCalculationServlet#Guinea-Bissau) | 85.36% | 3.5 | 0.68 |
| [Guinea-Bissau Black](http://tools.immuneepitope.org/tools/population/PopulationCalculationServlet#Guinea-Bissau%20Black) | 85.36% | 3.5 | 0.68 |
| [Russia Caucasoid](http://tools.immuneepitope.org/tools/population/PopulationCalculationServlet#Russia%20Caucasoid) | 84.51% | 10.44 | 1.29 |
| [Sao Tome and Principe](http://tools.immuneepitope.org/tools/population/PopulationCalculationServlet#Sao%20Tome%20and%20Principe) | 82.97% | 3.86 | 0.59 |
| [Sao Tome and Principe Black](http://tools.immuneepitope.org/tools/population/PopulationCalculationServlet#Sao%20Tome%20and%20Principe%20Black) | 82.97% | 3.86 | 0.59 |
| [Indonesia](http://tools.immuneepitope.org/tools/population/PopulationCalculationServlet#Indonesia) | 80.89% | 5.32 | 0.52 |
| [Indonesia Austronesian](http://tools.immuneepitope.org/tools/population/PopulationCalculationServlet#Indonesia%20Austronesian) | 80.89% | 5.32 | 0.52 |
| [Malaysia](http://tools.immuneepitope.org/tools/population/PopulationCalculationServlet#Malaysia) | 80.43% | 4.27 | 0.51 |
| [Switzerland](http://tools.immuneepitope.org/tools/population/PopulationCalculationServlet#Switzerland) | 80.29% | 11.86 | 2.03 |
| [Switzerland Caucasoid](http://tools.immuneepitope.org/tools/population/PopulationCalculationServlet#Switzerland%20Caucasoid) | 80.29% | 11.86 | 2.03 |
| [South Africa Black](http://tools.immuneepitope.org/tools/population/PopulationCalculationServlet#South%20Africa%20Black) | 79.90% | 3.07 | 0.5 |
| [Sudan Arab](http://tools.immuneepitope.org/tools/population/PopulationCalculationServlet#Sudan%20Arab) | 78.45% | 4.07 | 0.46 |
| [Ecuador](http://tools.immuneepitope.org/tools/population/PopulationCalculationServlet#Ecuador) | 76.97% | 1.48 | 0.43 |
| [Ecuador Amerindian](http://tools.immuneepitope.org/tools/population/PopulationCalculationServlet#Ecuador%20Amerindian) | 76.97% | 1.48 | 0.43 |
| [Russia Mixed](http://tools.immuneepitope.org/tools/population/PopulationCalculationServlet#Russia%20Mixed) | 76.89% | 11.26 | 0.87 |
| [Lebanon](http://tools.immuneepitope.org/tools/population/PopulationCalculationServlet#Lebanon) | 72.23% | 9.87 | 1.44 |
| [Lebanon Mixed](http://tools.immuneepitope.org/tools/population/PopulationCalculationServlet#Lebanon%20Mixed) | 72.23% | 9.87 | 1.44 |
| [Turkey](http://tools.immuneepitope.org/tools/population/PopulationCalculationServlet#Turkey) | 71.46% | 8.58 | 1.4 |
| [Turkey Caucasoid](http://tools.immuneepitope.org/tools/population/PopulationCalculationServlet#Turkey%20Caucasoid) | 71.46% | 8.58 | 1.4 |
| [Malaysia Austronesian](http://tools.immuneepitope.org/tools/population/PopulationCalculationServlet#Malaysia%20Austronesian) | 71.10% | 4.95 | 0.35 |
| [Burkina Faso](http://tools.immuneepitope.org/tools/population/PopulationCalculationServlet#Burkina%20Faso) | 69.55% | 5.09 | 0.33 |
| [Burkina Faso Black](http://tools.immuneepitope.org/tools/population/PopulationCalculationServlet#Burkina%20Faso%20Black) | 69.55% | 5.09 | 0.33 |
| Average | **59.25%** | **6.07** | **?** |
| [Scotland](http://tools.immuneepitope.org/tools/population/PopulationCalculationServlet#Scotland) | 50.68% | 7.41 | 0.41 |
| [Scotland Caucasoid](http://tools.immuneepitope.org/tools/population/PopulationCalculationServlet#Scotland%20Caucasoid) | 50.68% | 7.41 | 0.41 |
| [Sri Lanka](http://tools.immuneepitope.org/tools/population/PopulationCalculationServlet#Sri%20Lanka) | 49.02% | 1.56 | 0.2 |
| [Sri Lanka Asian](http://tools.immuneepitope.org/tools/population/PopulationCalculationServlet#Sri%20Lanka%20Asian) | 49.02% | 1.56 | 0.2 |
| [England Jew](http://tools.immuneepitope.org/tools/population/PopulationCalculationServlet#England%20Jew) | 47.87% | 3.24 | 0.77 |
| [Rwanda](http://tools.immuneepitope.org/tools/population/PopulationCalculationServlet#Rwanda) | 36.90% | 1.48 | 0.32 |
| [Rwanda Black](http://tools.immuneepitope.org/tools/population/PopulationCalculationServlet#Rwanda%20Black) | 36.90% | 1.48 | 0.32 |
| [Macedonia](http://tools.immuneepitope.org/tools/population/PopulationCalculationServlet#Macedonia) | 31.87% | 2.46 | 0.15 |
| [Macedonia Caucasoid](http://tools.immuneepitope.org/tools/population/PopulationCalculationServlet#Macedonia%20Caucasoid) | 31.87% | 2.46 | 0.15 |
| [Serbia](http://tools.immuneepitope.org/tools/population/PopulationCalculationServlet#Serbia) | 29.78% | 0.43 | 0.14 |
| [Serbia Caucasoid](http://tools.immuneepitope.org/tools/population/PopulationCalculationServlet#Serbia%20Caucasoid) | 29.78% | 0.43 | 0.14 |
| [Martinique](http://tools.immuneepitope.org/tools/population/PopulationCalculationServlet#Martinique) | 22.56% | 0.68 | 0.39 |
| [Martinique Black](http://tools.immuneepitope.org/tools/population/PopulationCalculationServlet#Martinique%20Black) | 22.56% | 0.68 | 0.39 |
| [Central African Republic](http://tools.immuneepitope.org/tools/population/PopulationCalculationServlet#Central%20African%20Republic) | 18.71% | 0.61 | 0.12 |
| [Central African Republic Black](http://tools.immuneepitope.org/tools/population/PopulationCalculationServlet#Central%20African%20Republic%20Black) | 18.71% | 0.61 | 0.12 |
| [Ivory Coast](http://tools.immuneepitope.org/tools/population/PopulationCalculationServlet#Ivory%20Coast) | 15.33% | 0.16 | 0.12 |
| [Ivory Coast Black](http://tools.immuneepitope.org/tools/population/PopulationCalculationServlet#Ivory%20Coast%20Black) | 15.33% | 0.16 | 0.12 |
| [Colombia Mestizo](http://tools.immuneepitope.org/tools/population/PopulationCalculationServlet#Colombia%20Mestizo) | 14.07% | 0.57 | 0.47 |
| [United Arab Emirates](http://tools.immuneepitope.org/tools/population/PopulationCalculationServlet#United%20Arab%20Emirates) | 12.20% | 0.36 | 0.11 |
| [United Arab Emirates Arab](http://tools.immuneepitope.org/tools/population/PopulationCalculationServlet#United%20Arab%20Emirates%20Arab) | 12.20% | 0.36 | 0.11 |
| [Equatorial Guinea](http://tools.immuneepitope.org/tools/population/PopulationCalculationServlet#Equatorial%20Guinea) | 9.75% | 2.51 | 2.44 |
| [Equatorial Guinea Black](http://tools.immuneepitope.org/tools/population/PopulationCalculationServlet#Equatorial%20Guinea%20Black) | 9.75% | 2.51 | 2.44 |
| [Venezuela Caucasoid](http://tools.immuneepitope.org/tools/population/PopulationCalculationServlet#Venezuela%20Caucasoid) | 9.18% | 0.28 | 0.33 |
| [Colombia](http://tools.immuneepitope.org/tools/population/PopulationCalculationServlet#Colombia) | 8.36% | 0.34 | 0.44 |
| [Venezuela Mestizo](http://tools.immuneepitope.org/tools/population/PopulationCalculationServlet#Venezuela%20Mestizo) | 7.84% | 0.24 | 0.33 |
| [Colombia Black](http://tools.immuneepitope.org/tools/population/PopulationCalculationServlet#Colombia%20Black) | 3.65% | 0.15 | 0.42 |
| [Central America](http://tools.immuneepitope.org/tools/population/PopulationCalculationServlet#Central%20America) | 3.55% | 0.08 | 0.1 |
| [Guatemala](http://tools.immuneepitope.org/tools/population/PopulationCalculationServlet#Guatemala) | 3.55% | 0.08 | 0.1 |
| [Guatemala Amerindian](http://tools.immuneepitope.org/tools/population/PopulationCalculationServlet#Guatemala%20Amerindian) | 3.55% | 0.08 | 0.1 |
| [Peru Mestizo](http://tools.immuneepitope.org/tools/population/PopulationCalculationServlet#Peru%20Mestizo) | 1.99% | 0.44 | 2.24 |
| [Wales](http://tools.immuneepitope.org/tools/population/PopulationCalculationServlet#Wales) | 1.00% | 0.01 | 0.1 |
| [Wales Caucasoid](http://tools.immuneepitope.org/tools/population/PopulationCalculationServlet#Wales%20Caucasoid) | 1.00% | 0.01 | 0.1 |

**^a^** projected population coverage 
**^b^** average number of epitope hits / HLA combinations recognized by the population 
**^c^** minimum number of epitope hits / HLA combinations recognized by 90% of the population

*For Glycoprotein (G)*

| Population / Area | **Class I** | | |
| --- | --- | --- | --- |
|  | **Coverage ^a^** | **Average hit ^b^** | **PC90 ^c^** |
| [World](http://tools.immuneepitope.org/tools/population/PopulationCalculationServlet#World) | 91.93% | 5.59 | 1.39 |
| [Finland](http://tools.immuneepitope.org/tools/population/PopulationCalculationServlet#Finland) | 98.84% | 7.65 | 2.74 |
| [Finland Caucasoid](http://tools.immuneepitope.org/tools/population/PopulationCalculationServlet#Finland%20Caucasoid) | 98.84% | 7.65 | 2.74 |
| [Mexico Amerindian](http://tools.immuneepitope.org/tools/population/PopulationCalculationServlet#Mexico%20Amerindian) | 97.39% | 5.5 | 2.4 |
| [Philippines](http://tools.immuneepitope.org/tools/population/PopulationCalculationServlet#Philippines) | 96.70% | 7.86 | 2.1 |
| [Philippines Austronesian](http://tools.immuneepitope.org/tools/population/PopulationCalculationServlet#Philippines%20Austronesian) | 96.70% | 7.86 | 2.1 |
| [United States Polynesian](http://tools.immuneepitope.org/tools/population/PopulationCalculationServlet#United%20States%20Polynesian) | 96.65% | 9.12 | 2.37 |
| [Germany](http://tools.immuneepitope.org/tools/population/PopulationCalculationServlet#Germany) | 96.41% | 6.34 | 2.2 |
| [Germany Caucasoid](http://tools.immuneepitope.org/tools/population/PopulationCalculationServlet#Germany%20Caucasoid) | 96.41% | 6.34 | 2.2 |
| [Australia Caucasoid](http://tools.immuneepitope.org/tools/population/PopulationCalculationServlet#Australia%20Caucasoid) | 95.69% | 4.99 | 2.09 |
| [Ireland Northern](http://tools.immuneepitope.org/tools/population/PopulationCalculationServlet#Ireland%20Northern) | 95.65% | 5.25 | 2.1 |
| [Ireland Northern Caucasoid](http://tools.immuneepitope.org/tools/population/PopulationCalculationServlet#Ireland%20Northern%20Caucasoid) | 95.65% | 5.25 | 2.1 |
| [Russia Other](http://tools.immuneepitope.org/tools/population/PopulationCalculationServlet#Russia%20Other) | 95.58% | 5.98 | 1.91 |
| [England Caucasoid](http://tools.immuneepitope.org/tools/population/PopulationCalculationServlet#England%20Caucasoid) | 95.48% | 6.05 | 2.11 |
| [United States Caucasoid](http://tools.immuneepitope.org/tools/population/PopulationCalculationServlet#United%20States%20Caucasoid) | 95.05% | 5.96 | 2.07 |
| [Poland](http://tools.immuneepitope.org/tools/population/PopulationCalculationServlet#Poland) | 94.79% | 6.57 | 2.07 |
| [Poland Caucasoid](http://tools.immuneepitope.org/tools/population/PopulationCalculationServlet#Poland%20Caucasoid) | 94.79% | 6.57 | 2.07 |
| [Taiwan](http://tools.immuneepitope.org/tools/population/PopulationCalculationServlet#Taiwan) | 94.42% | 5.62 | 1.6 |
| [Taiwan Oriental](http://tools.immuneepitope.org/tools/population/PopulationCalculationServlet#Taiwan%20Oriental) | 94.42% | 5.62 | 1.6 |
| [Mexico](http://tools.immuneepitope.org/tools/population/PopulationCalculationServlet#Mexico) | 94.40% | 4.98 | 2.05 |
| [Sweden](http://tools.immuneepitope.org/tools/population/PopulationCalculationServlet#Sweden) | 94.39% | 3.25 | 1.9 |
| [Sweden Caucasoid](http://tools.immuneepitope.org/tools/population/PopulationCalculationServlet#Sweden%20Caucasoid) | 94.39% | 3.25 | 1.9 |
| [Europe](http://tools.immuneepitope.org/tools/population/PopulationCalculationServlet#Europe) | 94.38% | 6.11 | 2.03 |
| [France](http://tools.immuneepitope.org/tools/population/PopulationCalculationServlet#France) | 94.18% | 6.11 | 1.88 |
| [France Caucasoid](http://tools.immuneepitope.org/tools/population/PopulationCalculationServlet#France%20Caucasoid) | 94.18% | 6.11 | 1.88 |
| [Venezuela](http://tools.immuneepitope.org/tools/population/PopulationCalculationServlet#Venezuela) | 94.03% | 3.53 | 1.9 |
| [Bulgaria](http://tools.immuneepitope.org/tools/population/PopulationCalculationServlet#Bulgaria) | 93.84% | 4.93 | 2.05 |
| [Venezuela Amerindian](http://tools.immuneepitope.org/tools/population/PopulationCalculationServlet#Venezuela%20Amerindian) | 93.76% | 3.51 | 1.91 |
| [Georgia Caucasoid](http://tools.immuneepitope.org/tools/population/PopulationCalculationServlet#Georgia%20Caucasoid) | 93.57% | 6.89 | 2.01 |
| [Ireland South](http://tools.immuneepitope.org/tools/population/PopulationCalculationServlet#Ireland%20South) | 93.45% | 5.03 | 1.71 |
| [Ireland South Caucasoid](http://tools.immuneepitope.org/tools/population/PopulationCalculationServlet#Ireland%20South%20Caucasoid) | 93.45% | 5.03 | 1.71 |
| [Italy](http://tools.immuneepitope.org/tools/population/PopulationCalculationServlet#Italy) | 93.28% | 6.97 | 2 |
| [Italy Caucasoid](http://tools.immuneepitope.org/tools/population/PopulationCalculationServlet#Italy%20Caucasoid) | 93.28% | 6.97 | 2 |
| [United States Amerindian](http://tools.immuneepitope.org/tools/population/PopulationCalculationServlet#United%20States%20Amerindian) | 93.06% | 4.41 | 1.48 |
| [Czech Republic](http://tools.immuneepitope.org/tools/population/PopulationCalculationServlet#Czech%20Republic) | 93.05% | 4.02 | 1.81 |
| [Czech Republic Caucasoid](http://tools.immuneepitope.org/tools/population/PopulationCalculationServlet#Czech%20Republic%20Caucasoid) | 93.05% | 4.02 | 1.81 |
| [England](http://tools.immuneepitope.org/tools/population/PopulationCalculationServlet#England) | 92.73% | 5.03 | 1.71 |
| [Russia Siberian](http://tools.immuneepitope.org/tools/population/PopulationCalculationServlet#Russia%20Siberian) | 92.70% | 6.12 | 1.52 |
| [Korea; South](http://tools.immuneepitope.org/tools/population/PopulationCalculationServlet#Korea;%20South) | 92.47% | 6.93 | 1.59 |
| [Korea; South Oriental](http://tools.immuneepitope.org/tools/population/PopulationCalculationServlet#Korea;%20South%20Oriental) | 92.47% | 6.93 | 1.59 |
| [Iran](http://tools.immuneepitope.org/tools/population/PopulationCalculationServlet#Iran) | 92.40% | 5.6 | 2.01 |
| [Iran Persian](http://tools.immuneepitope.org/tools/population/PopulationCalculationServlet#Iran%20Persian) | 92.40% | 5.6 | 2.01 |
| [Russia](http://tools.immuneepitope.org/tools/population/PopulationCalculationServlet#Russia) | 92.39% | 5.95 | 1.45 |
| [United States](http://tools.immuneepitope.org/tools/population/PopulationCalculationServlet#United%20States) | 92.39% | 4.95 | 1.4 |
| [North America](http://tools.immuneepitope.org/tools/population/PopulationCalculationServlet#North%20America) | 92.31% | 4.91 | 1.38 |
| [United States Asian](http://tools.immuneepitope.org/tools/population/PopulationCalculationServlet#United%20States%20Asian) | 92.28% | 6.35 | 1.55 |
| [Austria](http://tools.immuneepitope.org/tools/population/PopulationCalculationServlet#Austria) | 92.09% | 2.87 | 1.45 |
| [Austria Caucasoid](http://tools.immuneepitope.org/tools/population/PopulationCalculationServlet#Austria%20Caucasoid) | 92.09% | 2.87 | 1.45 |
| [United States Mestizo](http://tools.immuneepitope.org/tools/population/PopulationCalculationServlet#United%20States%20Mestizo) | 92.02% | 5.18 | 1.36 |
| [Georgia](http://tools.immuneepitope.org/tools/population/PopulationCalculationServlet#Georgia) | 91.86% | 6.24 | 1.45 |
| [United States Hispanic](http://tools.immuneepitope.org/tools/population/PopulationCalculationServlet#United%20States%20Hispanic) | 91.72% | 4.97 | 1.27 |
| [Northeast Asia](http://tools.immuneepitope.org/tools/population/PopulationCalculationServlet#Northeast%20Asia) | 91.48% | 6.36 | 1.43 |
| [Peru](http://tools.immuneepitope.org/tools/population/PopulationCalculationServlet#Peru) | 91.15% | 2.58 | 1.14 |
| [China](http://tools.immuneepitope.org/tools/population/PopulationCalculationServlet#China) | 91.04% | 6.22 | 1.31 |
| [China Oriental](http://tools.immuneepitope.org/tools/population/PopulationCalculationServlet#China%20Oriental) | 91.04% | 6.22 | 1.31 |
| [Peru Amerindian](http://tools.immuneepitope.org/tools/population/PopulationCalculationServlet#Peru%20Amerindian) | 90.97% | 2.36 | 1.11 |
| [Australia](http://tools.immuneepitope.org/tools/population/PopulationCalculationServlet#Australia) | 90.93% | 5.58 | 1.12 |
| [Japan](http://tools.immuneepitope.org/tools/population/PopulationCalculationServlet#Japan) | 90.86% | 6.59 | 1.15 |
| [Japan Oriental](http://tools.immuneepitope.org/tools/population/PopulationCalculationServlet#Japan%20Oriental) | 90.86% | 6.59 | 1.15 |
| [Singapore Oriental](http://tools.immuneepitope.org/tools/population/PopulationCalculationServlet#Singapore%20Oriental) | 90.84% | 5.15 | 1.17 |
| [Mali](http://tools.immuneepitope.org/tools/population/PopulationCalculationServlet#Mali) | 90.76% | 3.31 | 1.03 |
| [Mali Black](http://tools.immuneepitope.org/tools/population/PopulationCalculationServlet#Mali%20Black) | 90.76% | 3.31 | 1.03 |
| [Papua New Guinea](http://tools.immuneepitope.org/tools/population/PopulationCalculationServlet#Papua%20New%20Guinea) | 90.73% | 5.38 | 1.09 |
| [Papua New Guinea Melanesian](http://tools.immuneepitope.org/tools/population/PopulationCalculationServlet#Papua%20New%20Guinea%20Melanesian) | 90.73% | 5.38 | 1.09 |
| [Southeast Asia](http://tools.immuneepitope.org/tools/population/PopulationCalculationServlet#Southeast%20Asia) | 90.44% | 5.25 | 1.06 |
| [American Samoa](http://tools.immuneepitope.org/tools/population/PopulationCalculationServlet#American%20Samoa) | 90.36% | 3.91 | 1.03 |
| [American Samoa Polynesian](http://tools.immuneepitope.org/tools/population/PopulationCalculationServlet#American%20Samoa%20Polynesian) | 90.36% | 3.91 | 1.03 |
| [Bulgaria Other](http://tools.immuneepitope.org/tools/population/PopulationCalculationServlet#Bulgaria%20Other) | 90.13% | 4.57 | 1.13 |
| [East Asia](http://tools.immuneepitope.org/tools/population/PopulationCalculationServlet#East%20Asia) | 90.06% | 6.47 | 1.01 |
| [Portugal](http://tools.immuneepitope.org/tools/population/PopulationCalculationServlet#Portugal) | 89.60% | 4.98 | 0.96 |
| [Portugal Caucasoid](http://tools.immuneepitope.org/tools/population/PopulationCalculationServlet#Portugal%20Caucasoid) | 89.60% | 4.98 | 0.96 |
| [Australia Australian Aborigines](http://tools.immuneepitope.org/tools/population/PopulationCalculationServlet#Australia%20Australian%20Aborigines) | 88.84% | 5.38 | 0.9 |
| [Romania](http://tools.immuneepitope.org/tools/population/PopulationCalculationServlet#Romania) | 88.30% | 2.63 | 0.85 |
| [Romania Caucasoid](http://tools.immuneepitope.org/tools/population/PopulationCalculationServlet#Romania%20Caucasoid) | 88.30% | 2.63 | 0.85 |
| [Vietnam](http://tools.immuneepitope.org/tools/population/PopulationCalculationServlet#Vietnam) | 88.24% | 6.51 | 0.85 |
| [Vietnam Oriental](http://tools.immuneepitope.org/tools/population/PopulationCalculationServlet#Vietnam%20Oriental) | 88.24% | 6.51 | 0.85 |
| [Brazil Mixed](http://tools.immuneepitope.org/tools/population/PopulationCalculationServlet#Brazil%20Mixed) | 88.07% | 4.9 | 0.84 |
| [Saudi Arabia](http://tools.immuneepitope.org/tools/population/PopulationCalculationServlet#Saudi%20Arabia) | 87.98% | 4.22 | 0.83 |
| [Saudi Arabia Arab](http://tools.immuneepitope.org/tools/population/PopulationCalculationServlet#Saudi%20Arabia%20Arab) | 87.98% | 4.22 | 0.83 |
| [Chile Amerindian](http://tools.immuneepitope.org/tools/population/PopulationCalculationServlet#Chile%20Amerindian) | 87.82% | 2.93 | 0.82 |
| [Chile Amerindian](http://tools.immuneepitope.org/tools/population/PopulationCalculationServlet#Chile%20Amerindian) | 87.82% | 2.93 | 0.82 |
| [Thailand](http://tools.immuneepitope.org/tools/population/PopulationCalculationServlet#Thailand) | 87.66% | 5.29 | 0.81 |
| [Thailand Oriental](http://tools.immuneepitope.org/tools/population/PopulationCalculationServlet#Thailand%20Oriental) | 87.66% | 5.29 | 0.81 |
| [Hong Kong](http://tools.immuneepitope.org/tools/population/PopulationCalculationServlet#Hong%20Kong) | 87.43% | 4.07 | 0.8 |
| [Hong Kong Oriental](http://tools.immuneepitope.org/tools/population/PopulationCalculationServlet#Hong%20Kong%20Oriental) | 87.43% | 4.07 | 0.8 |
| [Croatia](http://tools.immuneepitope.org/tools/population/PopulationCalculationServlet#Croatia) | 87.41% | 2.56 | 0.79 |
| [Croatia Caucasoid](http://tools.immuneepitope.org/tools/population/PopulationCalculationServlet#Croatia%20Caucasoid) | 87.41% | 2.56 | 0.79 |
| [Brazil Amerindian](http://tools.immuneepitope.org/tools/population/PopulationCalculationServlet#Brazil%20Amerindian) | 87.33% | 3.87 | 0.79 |
| [Singapore](http://tools.immuneepitope.org/tools/population/PopulationCalculationServlet#Singapore) | 87.18% | 4.84 | 0.78 |
| [Pakistan Asian](http://tools.immuneepitope.org/tools/population/PopulationCalculationServlet#Pakistan%20Asian) | 86.95% | 4.45 | 0.77 |
| [Belgium](http://tools.immuneepitope.org/tools/population/PopulationCalculationServlet#Belgium) | 86.93% | 2.22 | 0.77 |
| [Belgium Caucasoid](http://tools.immuneepitope.org/tools/population/PopulationCalculationServlet#Belgium%20Caucasoid) | 86.93% | 2.22 | 0.77 |
| [Brazil](http://tools.immuneepitope.org/tools/population/PopulationCalculationServlet#Brazil) | 86.89% | 4.5 | 0.76 |
| [United States Black](http://tools.immuneepitope.org/tools/population/PopulationCalculationServlet#United%20States%20Black) | 86.88% | 3.31 | 0.76 |
| [Tunisia](http://tools.immuneepitope.org/tools/population/PopulationCalculationServlet#Tunisia) | 86.82% | 4.24 | 0.76 |
| [Tunisia Arab](http://tools.immuneepitope.org/tools/population/PopulationCalculationServlet#Tunisia%20Arab) | 86.82% | 4.24 | 0.76 |
| [Bulgaria Caucasoid](http://tools.immuneepitope.org/tools/population/PopulationCalculationServlet#Bulgaria%20Caucasoid) | 86.75% | 2.37 | 0.75 |
| [Israel Arab](http://tools.immuneepitope.org/tools/population/PopulationCalculationServlet#Israel%20Arab) | 86.61% | 4.36 | 0.75 |
| [Oman](http://tools.immuneepitope.org/tools/population/PopulationCalculationServlet#Oman) | 86.58% | 2.57 | 0.75 |
| [Oman Arab](http://tools.immuneepitope.org/tools/population/PopulationCalculationServlet#Oman%20Arab) | 86.58% | 2.57 | 0.75 |
| [Georgia Kurd](http://tools.immuneepitope.org/tools/population/PopulationCalculationServlet#Georgia%20Kurd) | 86.36% | 4.56 | 0.73 |
| [Spain](http://tools.immuneepitope.org/tools/population/PopulationCalculationServlet#Spain) | 86.28% | 5.49 | 0.73 |
| [Spain Caucasoid](http://tools.immuneepitope.org/tools/population/PopulationCalculationServlet#Spain%20Caucasoid) | 86.28% | 5.49 | 0.73 |
| [Sudan Mixed](http://tools.immuneepitope.org/tools/population/PopulationCalculationServlet#Sudan%20Mixed) | 86.21% | 5.56 | 0.73 |
| [Pakistan](http://tools.immuneepitope.org/tools/population/PopulationCalculationServlet#Pakistan) | 86.10% | 4.19 | 0.72 |
| [Oceania](http://tools.immuneepitope.org/tools/population/PopulationCalculationServlet#Oceania) | 86.00% | 4.52 | 0.71 |
| [Morocco Caucasoid](http://tools.immuneepitope.org/tools/population/PopulationCalculationServlet#Morocco%20Caucasoid) | 85.99% | 3.44 | 0.71 |
| [Cape Verde](http://tools.immuneepitope.org/tools/population/PopulationCalculationServlet#Cape%20Verde) | 85.85% | 2.44 | 0.71 |
| [Cape Verde Black](http://tools.immuneepitope.org/tools/population/PopulationCalculationServlet#Cape%20Verde%20Black) | 85.85% | 2.44 | 0.71 |
| [Chile](http://tools.immuneepitope.org/tools/population/PopulationCalculationServlet#Chile) | 85.76% | 3.95 | 0.7 |
| [Chile](http://tools.immuneepitope.org/tools/population/PopulationCalculationServlet#Chile) | 85.76% | 3.95 | 0.7 |
| [North Africa](http://tools.immuneepitope.org/tools/population/PopulationCalculationServlet#North%20Africa) | 85.69% | 3.95 | 0.7 |
| [Sudan](http://tools.immuneepitope.org/tools/population/PopulationCalculationServlet#Sudan) | 85.53% | 5.16 | 0.69 |
| [South Asia](http://tools.immuneepitope.org/tools/population/PopulationCalculationServlet#South%20Asia) | 85.19% | 4.74 | 0.68 |
| [Cuba Mulatto](http://tools.immuneepitope.org/tools/population/PopulationCalculationServlet#Cuba%20Mulatto) | 84.59% | 2.33 | 0.65 |
| [Morocco](http://tools.immuneepitope.org/tools/population/PopulationCalculationServlet#Morocco) | 84.49% | 3.04 | 0.64 |
| [Guinea-Bissau](http://tools.immuneepitope.org/tools/population/PopulationCalculationServlet#Guinea-Bissau) | 84.43% | 2.24 | 0.64 |
| [Guinea-Bissau Black](http://tools.immuneepitope.org/tools/population/PopulationCalculationServlet#Guinea-Bissau%20Black) | 84.43% | 2.24 | 0.64 |
| [Pakistan Mixed](http://tools.immuneepitope.org/tools/population/PopulationCalculationServlet#Pakistan%20Mixed) | 84.02% | 3.61 | 0.63 |
| [Brazil Caucasoid](http://tools.immuneepitope.org/tools/population/PopulationCalculationServlet#Brazil%20Caucasoid) | 83.31% | 2.09 | 0.6 |
| [South Africa](http://tools.immuneepitope.org/tools/population/PopulationCalculationServlet#South%20Africa) | 83.04% | 4.03 | 0.59 |
| [South Africa](http://tools.immuneepitope.org/tools/population/PopulationCalculationServlet#South%20Africa) | 83.04% | 4.03 | 0.59 |
| [West Africa](http://tools.immuneepitope.org/tools/population/PopulationCalculationServlet#West%20Africa) | 82.86% | 2.69 | 0.58 |
| [Mexico Mestizo](http://tools.immuneepitope.org/tools/population/PopulationCalculationServlet#Mexico%20Mestizo) | 82.71% | 2.47 | 0.58 |
| [Singapore Austronesian](http://tools.immuneepitope.org/tools/population/PopulationCalculationServlet#Singapore%20Austronesian) | 82.61% | 4.74 | 0.58 |
| [Morocco Arab](http://tools.immuneepitope.org/tools/population/PopulationCalculationServlet#Morocco%20Arab) | 82.50% | 2.5 | 0.57 |
| [Uganda](http://tools.immuneepitope.org/tools/population/PopulationCalculationServlet#Uganda) | 82.21% | 3.37 | 0.56 |
| [Uganda Black](http://tools.immuneepitope.org/tools/population/PopulationCalculationServlet#Uganda%20Black) | 82.21% | 3.37 | 0.56 |
| [Senegal](http://tools.immuneepitope.org/tools/population/PopulationCalculationServlet#Senegal) | 82.06% | 2.56 | 0.56 |
| [Senegal Black](http://tools.immuneepitope.org/tools/population/PopulationCalculationServlet#Senegal%20Black) | 82.06% | 2.56 | 0.56 |
| [West Indies](http://tools.immuneepitope.org/tools/population/PopulationCalculationServlet#West%20Indies) | 82.02% | 2.15 | 0.56 |
| [Cuba](http://tools.immuneepitope.org/tools/population/PopulationCalculationServlet#Cuba) | 81.32% | 2.14 | 0.54 |
| [Chile Mixed](http://tools.immuneepitope.org/tools/population/PopulationCalculationServlet#Chile%20Mixed) | 80.98% | 3.23 | 0.53 |
| [Southwest Asia](http://tools.immuneepitope.org/tools/population/PopulationCalculationServlet#Southwest%20Asia) | 80.20% | 3.7 | 0.51 |
| [Sao Tome and Principe](http://tools.immuneepitope.org/tools/population/PopulationCalculationServlet#Sao%20Tome%20and%20Principe) | 79.41% | 1.93 | 0.49 |
| [Sao Tome and Principe Black](http://tools.immuneepitope.org/tools/population/PopulationCalculationServlet#Sao%20Tome%20and%20Principe%20Black) | 79.41% | 1.93 | 0.49 |
| [South Africa Other](http://tools.immuneepitope.org/tools/population/PopulationCalculationServlet#South%20Africa%20Other) | 79.41% | 4.24 | 0.49 |
| [Cuba Caucasoid](http://tools.immuneepitope.org/tools/population/PopulationCalculationServlet#Cuba%20Caucasoid) | 79.26% | 2.02 | 0.48 |
| [South America](http://tools.immuneepitope.org/tools/population/PopulationCalculationServlet#South%20America) | 79.10% | 3.2 | 0.48 |
| [India](http://tools.immuneepitope.org/tools/population/PopulationCalculationServlet#India) | 78.80% | 4.29 | 0.47 |
| [India Asian](http://tools.immuneepitope.org/tools/population/PopulationCalculationServlet#India%20Asian) | 78.80% | 4.29 | 0.47 |
| [Malaysia Oriental](http://tools.immuneepitope.org/tools/population/PopulationCalculationServlet#Malaysia%20Oriental) | 78.37% | 2.59 | 0.46 |
| [Zambia](http://tools.immuneepitope.org/tools/population/PopulationCalculationServlet#Zambia) | 78.33% | 2.25 | 0.46 |
| [Zambia Black](http://tools.immuneepitope.org/tools/population/PopulationCalculationServlet#Zambia%20Black) | 78.33% | 2.25 | 0.46 |
| [Zimbabwe](http://tools.immuneepitope.org/tools/population/PopulationCalculationServlet#Zimbabwe) | 78.22% | 2.04 | 0.46 |
| [Zimbabwe Black](http://tools.immuneepitope.org/tools/population/PopulationCalculationServlet#Zimbabwe%20Black) | 78.22% | 2.04 | 0.46 |
| [Argentina](http://tools.immuneepitope.org/tools/population/PopulationCalculationServlet#Argentina) | 77.87% | 1.71 | 0.45 |
| [Argentina Amerindian](http://tools.immuneepitope.org/tools/population/PopulationCalculationServlet#Argentina%20Amerindian) | 77.87% | 1.71 | 0.45 |
| [Jordan](http://tools.immuneepitope.org/tools/population/PopulationCalculationServlet#Jordan) | 76.11% | 3.08 | 0.42 |
| [Jordan Arab](http://tools.immuneepitope.org/tools/population/PopulationCalculationServlet#Jordan%20Arab) | 76.11% | 3.08 | 0.42 |
| [Cameroon](http://tools.immuneepitope.org/tools/population/PopulationCalculationServlet#Cameroon) | 75.98% | 2.74 | 0.42 |
| [Cameroon Black](http://tools.immuneepitope.org/tools/population/PopulationCalculationServlet#Cameroon%20Black) | 75.98% | 2.74 | 0.42 |
| [New Caledonia](http://tools.immuneepitope.org/tools/population/PopulationCalculationServlet#New%20Caledonia) | 75.25% | 3.13 | 0.4 |
| [New Caledonia Melanesian](http://tools.immuneepitope.org/tools/population/PopulationCalculationServlet#New%20Caledonia%20Melanesian) | 75.25% | 3.13 | 0.4 |
| [East Africa](http://tools.immuneepitope.org/tools/population/PopulationCalculationServlet#East%20Africa) | 74.93% | 2.35 | 0.4 |
| [Israel](http://tools.immuneepitope.org/tools/population/PopulationCalculationServlet#Israel) | 74.46% | 3 | 0.39 |
| [Kenya](http://tools.immuneepitope.org/tools/population/PopulationCalculationServlet#Kenya) | 74.23% | 2.19 | 0.39 |
| [Kenya Black](http://tools.immuneepitope.org/tools/population/PopulationCalculationServlet#Kenya%20Black) | 74.23% | 2.19 | 0.39 |
| [Malaysia](http://tools.immuneepitope.org/tools/population/PopulationCalculationServlet#Malaysia) | 73.33% | 2.69 | 0.37 |
| [Central Africa](http://tools.immuneepitope.org/tools/population/PopulationCalculationServlet#Central%20Africa) | 72.82% | 2.72 | 0.37 |
| [South Africa Black](http://tools.immuneepitope.org/tools/population/PopulationCalculationServlet#South%20Africa%20Black) | 72.80% | 1.56 | 0.37 |
| [Israel Jew](http://tools.immuneepitope.org/tools/population/PopulationCalculationServlet#Israel%20Jew) | 71.15% | 2.55 | 0.35 |
| [Indonesia](http://tools.immuneepitope.org/tools/population/PopulationCalculationServlet#Indonesia) | 70.30% | 3.5 | 0.34 |
| [Indonesia Austronesian](http://tools.immuneepitope.org/tools/population/PopulationCalculationServlet#Indonesia%20Austronesian) | 70.30% | 3.5 | 0.34 |
| [Malaysia Austronesian](http://tools.immuneepitope.org/tools/population/PopulationCalculationServlet#Malaysia%20Austronesian) | 68.28% | 3.75 | 0.32 |
| [Russia Mixed](http://tools.immuneepitope.org/tools/population/PopulationCalculationServlet#Russia%20Mixed) | 65.89% | 4.4 | 0.29 |
| [Russia Caucasoid](http://tools.immuneepitope.org/tools/population/PopulationCalculationServlet#Russia%20Caucasoid) | 62.25% | 3.18 | 0.26 |
| [Turkey](http://tools.immuneepitope.org/tools/population/PopulationCalculationServlet#Turkey) | 60.86% | 3.3 | 0.51 |
| [Turkey Caucasoid](http://tools.immuneepitope.org/tools/population/PopulationCalculationServlet#Turkey%20Caucasoid) | 60.86% | 3.3 | 0.51 |
| [United Kingdom](http://tools.immuneepitope.org/tools/population/PopulationCalculationServlet#United%20Kingdom) | 60.56% | 2.86 | 0.25 |
| [United Kingdom Caucasoid](http://tools.immuneepitope.org/tools/population/PopulationCalculationServlet#United%20Kingdom%20Caucasoid) | 60.56% | 2.86 | 0.25 |
| [Switzerland](http://tools.immuneepitope.org/tools/population/PopulationCalculationServlet#Switzerland) | 59.55% | 4.14 | 0.49 |
| [Switzerland Caucasoid](http://tools.immuneepitope.org/tools/population/PopulationCalculationServlet#Switzerland%20Caucasoid) | 59.55% | 4.14 | 0.49 |
| [Mongolia](http://tools.immuneepitope.org/tools/population/PopulationCalculationServlet#Mongolia) | 56.13% | 1.29 | 0.23 |
| [Mongolia Oriental](http://tools.immuneepitope.org/tools/population/PopulationCalculationServlet#Mongolia%20Oriental) | 56.13% | 1.29 | 0.23 |
| [Sudan Arab](http://tools.immuneepitope.org/tools/population/PopulationCalculationServlet#Sudan%20Arab) | 56.08% | 1.4 | 0.23 |
| Average | **53.09%** | **2.58** | **?** |
| [Burkina Faso](http://tools.immuneepitope.org/tools/population/PopulationCalculationServlet#Burkina%20Faso) | 52.03% | 1.51 | 0.21 |
| [Burkina Faso Black](http://tools.immuneepitope.org/tools/population/PopulationCalculationServlet#Burkina%20Faso%20Black) | 52.03% | 1.51 | 0.21 |
| [Scotland](http://tools.immuneepitope.org/tools/population/PopulationCalculationServlet#Scotland) | 45.71% | 3.41 | 0.18 |
| [Scotland Caucasoid](http://tools.immuneepitope.org/tools/population/PopulationCalculationServlet#Scotland%20Caucasoid) | 45.71% | 3.41 | 0.18 |
| [Lebanon](http://tools.immuneepitope.org/tools/population/PopulationCalculationServlet#Lebanon) | 42.70% | 3.41 | 0.17 |
| [Lebanon Mixed](http://tools.immuneepitope.org/tools/population/PopulationCalculationServlet#Lebanon%20Mixed) | 42.70% | 3.41 | 0.17 |
| [Sri Lanka](http://tools.immuneepitope.org/tools/population/PopulationCalculationServlet#Sri%20Lanka) | 32.27% | 0.73 | 0.3 |
| [Sri Lanka Asian](http://tools.immuneepitope.org/tools/population/PopulationCalculationServlet#Sri%20Lanka%20Asian) | 32.27% | 0.73 | 0.3 |
| [Serbia](http://tools.immuneepitope.org/tools/population/PopulationCalculationServlet#Serbia) | 29.78% | 0.54 | 0.14 |
| [Serbia Caucasoid](http://tools.immuneepitope.org/tools/population/PopulationCalculationServlet#Serbia%20Caucasoid) | 29.78% | 0.54 | 0.14 |
| [Macedonia](http://tools.immuneepitope.org/tools/population/PopulationCalculationServlet#Macedonia) | 28.90% | 1.22 | 0.14 |
| [Macedonia Caucasoid](http://tools.immuneepitope.org/tools/population/PopulationCalculationServlet#Macedonia%20Caucasoid) | 28.90% | 1.22 | 0.14 |
| [Ecuador](http://tools.immuneepitope.org/tools/population/PopulationCalculationServlet#Ecuador) | 22.72% | 0.36 | 0.13 |
| [Ecuador Amerindian](http://tools.immuneepitope.org/tools/population/PopulationCalculationServlet#Ecuador%20Amerindian) | 22.72% | 0.36 | 0.13 |
| [Martinique](http://tools.immuneepitope.org/tools/population/PopulationCalculationServlet#Martinique) | 22.56% | 0.23 | 0.13 |
| [Martinique Black](http://tools.immuneepitope.org/tools/population/PopulationCalculationServlet#Martinique%20Black) | 22.56% | 0.23 | 0.13 |
| [Ivory Coast](http://tools.immuneepitope.org/tools/population/PopulationCalculationServlet#Ivory%20Coast) | 15.33% | 0.31 | 0.24 |
| [Ivory Coast Black](http://tools.immuneepitope.org/tools/population/PopulationCalculationServlet#Ivory%20Coast%20Black) | 15.33% | 0.31 | 0.24 |
| [Colombia Mestizo](http://tools.immuneepitope.org/tools/population/PopulationCalculationServlet#Colombia%20Mestizo) | 14.07% | 0.28 | 0.23 |
| [Rwanda](http://tools.immuneepitope.org/tools/population/PopulationCalculationServlet#Rwanda) | 13.51% | 0.14 | 0.12 |
| [Rwanda Black](http://tools.immuneepitope.org/tools/population/PopulationCalculationServlet#Rwanda%20Black) | 13.51% | 0.14 | 0.12 |
| [England Jew](http://tools.immuneepitope.org/tools/population/PopulationCalculationServlet#England%20Jew) | 12.76% | 0.47 | 0.11 |
| [United Arab Emirates](http://tools.immuneepitope.org/tools/population/PopulationCalculationServlet#United%20Arab%20Emirates) | 12.20% | 0.24 | 0.11 |
| [United Arab Emirates Arab](http://tools.immuneepitope.org/tools/population/PopulationCalculationServlet#United%20Arab%20Emirates%20Arab) | 12.20% | 0.24 | 0.11 |
| [Venezuela Caucasoid](http://tools.immuneepitope.org/tools/population/PopulationCalculationServlet#Venezuela%20Caucasoid) | 11.45% | 0.14 | 0.11 |
| [Equatorial Guinea](http://tools.immuneepitope.org/tools/population/PopulationCalculationServlet#Equatorial%20Guinea) | 9.75% | 1.17 | 1.22 |
| [Equatorial Guinea Black](http://tools.immuneepitope.org/tools/population/PopulationCalculationServlet#Equatorial%20Guinea%20Black) | 9.75% | 1.17 | 1.22 |
| [Venezuela Mestizo](http://tools.immuneepitope.org/tools/population/PopulationCalculationServlet#Venezuela%20Mestizo) | 9.75% | 0.12 | 0.11 |
| [Colombia](http://tools.immuneepitope.org/tools/population/PopulationCalculationServlet#Colombia) | 8.36% | 0.17 | 0.22 |
| [Central African Republic](http://tools.immuneepitope.org/tools/population/PopulationCalculationServlet#Central%20African%20Republic) | 5.48% | 0.11 | 0.21 |
| [Central African Republic Black](http://tools.immuneepitope.org/tools/population/PopulationCalculationServlet#Central%20African%20Republic%20Black) | 5.48% | 0.11 | 0.21 |
| [Colombia Black](http://tools.immuneepitope.org/tools/population/PopulationCalculationServlet#Colombia%20Black) | 3.65% | 0.07 | 0.21 |
| [Central America](http://tools.immuneepitope.org/tools/population/PopulationCalculationServlet#Central%20America) | 3.55% | 0.05 | 0.1 |
| [Guatemala](http://tools.immuneepitope.org/tools/population/PopulationCalculationServlet#Guatemala) | 3.55% | 0.05 | 0.1 |
| [Guatemala Amerindian](http://tools.immuneepitope.org/tools/population/PopulationCalculationServlet#Guatemala%20Amerindian) | 3.55% | 0.05 | 0.1 |
| [Peru Mestizo](http://tools.immuneepitope.org/tools/population/PopulationCalculationServlet#Peru%20Mestizo) | 1.99% | 0.22 | 1.12 |
| [Wales](http://tools.immuneepitope.org/tools/population/PopulationCalculationServlet#Wales) | 1.00% | 0.02 | 0.2 |
| [Wales Caucasoid](http://tools.immuneepitope.org/tools/population/PopulationCalculationServlet#Wales%20Caucasoid) | 1.00% | 0.02 | 0.2 |

**^a^** projected population coverage 
**^b^** average number of epitope hits / HLA combinations recognized by the population 
**^c^** minimum number of epitope hits / HLA combinations recognized by 90% of the population
